# Manipulation of nuclear architecture through CRISPR-mediated chromosomal looping

**DOI:** 10.1038/ncomms15993

**Published:** 2017-07-13

**Authors:** Stefanie L. Morgan, Natasha C. Mariano, Abel Bermudez, Nicole L. Arruda, Fangting Wu, Yunhai Luo, Gautam Shankar, Lin Jia, Huiling Chen, Ji-Fan Hu, Andrew R. Hoffman, Chiao-Chain Huang, Sharon J. Pitteri, Kevin C. Wang

**Affiliations:** 1 Department of Dermatology, Program in Epithelial Biology, Stanford University School of Medicine, Stanford, California 94305, USA; 2 Program in Cancer Biology, Stanford University School of Medicine, Stanford, California 94305, USA; 3 Canary Center for Cancer Early Detection, Department of Radiology, Stanford University School of Medicine, Palo Alto, California 94304, USA; 4 Bridgewater State University, Department of Biology, Bridgewater, Massachusetts 02325, USA; 5 System Biosciences, Palo Alto, California 94303, USA; 6 Veterans Affairs Healthcare System, Palo Alto, California 94304, USA; 7 Stem Cell and Cancer Center, First Affiliated Hospital, Jilin University, Changchun 130021, China; 8 Department of Endocrinology, Xiangya Hospital, Central South University, Changsha, Hunan 410008, China

## Abstract

Chromatin looping is key to gene regulation, yet no broadly applicable methods to selectively modify chromatin loops have been described. We have engineered a method for chromatin loop reorganization using CRISPR-dCas9 (CLOuD9) to selectively and reversibly establish chromatin loops. We demonstrate the power of this technology to selectively modulate gene expression at targeted loci.

## Abstract (web summary)

Chromatin looping plays an important role in gene regulation and the ability to manipulate loops would aid in understanding how this occurs. Here the authors present CLOuD9, a system that uses dimerized Cas9 complexes to selectively and reversibly establish chromatin loops.

There is growing appreciation that genome organization and the folding of chromatin within the nucleus are key determinants of gene expression programs^1^,^2^. Further, it has long been postulated that chromosomal contacts created by the three-dimensional organization of chromatin are critical in the regulation of gene expression^3^. This has been most thoroughly demonstrated at the human β-globin gene locus, where a locus control region (LCR) regulates the expression of the distant β-like globin genes through formation of a long-range chromatin loop^4^. Interestingly, while numerous long-range looping interactions have been identified with the advent of genomic technologies^5^,^6^, precisely how chromatin loops and the dynamic genome architecture contribute to the regulation of gene expression to affect cellular functions is not fully understood. Furthermore, whether chromatin looping is a cause or a consequence of gene activity remains unknown.

A significant challenge to obtaining a comprehensive understanding of the functional organization and formation of chromatin loops has been the availability of tools to study them. Although multiple groups have forcibly looped distant regulatory elements to influence gene expression^7^,^8^,^9^,^10^, the described methodologies often involve either gross alterations of the linear DNA sequence or are technically challenging and demand significant prior knowledge of the loop-mediating factors. While recent work with the β-globin locus suggested that gene expression requires the pre-existing establishment of the proper chromosomal architecture^10^, how chromatin dynamics specifically impact transcription apparatus and overall genome folding are central issues to resolve. Although there is growing appreciation for the interplay between looping and gene function, the mechanisms by which long-range chromosomal contacts are established or maintained with regulatory elements such as enhancers and promoters to bring about functional changes in gene expression remain unclear.

Through targeted modification of the existing clustered regularly interspaced short palindromic repeats (CRISPR)-CRISPR-associated protein 9 (Cas9) system components, we have developed a powerful tool for targeted, reversible chromatin loop reorganization using nuclease-deficient Cas9 (dCas9; CLOuD9), as a broadly applicable method that enables the forced juxtaposition of any two genomic loci. Here we show that modification of loop structure can reversibly alter gene expression, and provide evidence of a possible mechanism for the formation of stable *de novo* chromatin loops observed in development and cancer alike.

## Results

### CLOuD9 induces reversible β-globin promoter-LCR looping

**Figure 1:**
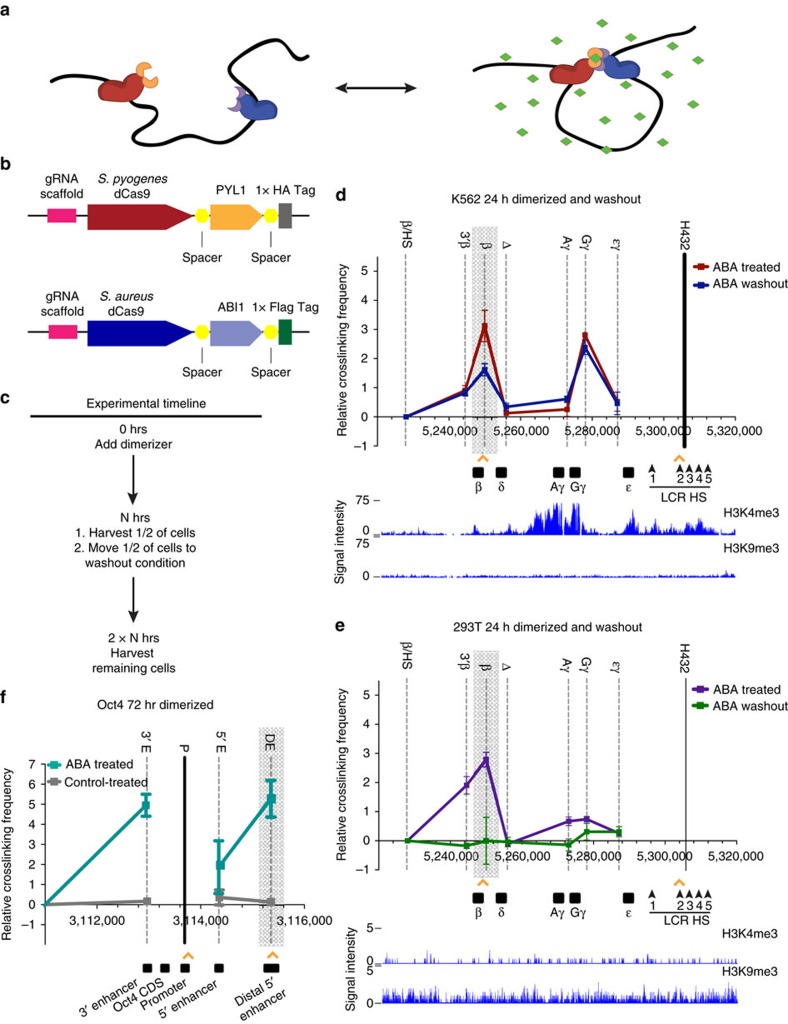
CLOuD9 induces reversible β-globin promoter-LCR looping. (**a**) Addition of abscisic acid (ABA, green) brings two complementary CLOuD9 constructs (CLOuD9 *S. pyogenes* (CSP), CLOuD9 *S. aureus* (CSA), red and blue, respectively) into proximity, remodelling chromatin structure. Removal of ABA restores the endogenous chromatin conformation. (**b**) CLOuD9 constructs combine CRISPR-dCas9 technology from *S. aureus* and *S. pyogenes* with reversibly dimerizeable PYL1 and ABI1 domains. (**c**) Timeline of CLOuD9 dimerization experiments. (**d**) 3C assay measuring β-globin locus-wide crosslinking frequencies in K562 cells after 24 h of treatment with ABA (red) and subsequent washout (blue) showing reversibility of induced β-globin/LCR contacts (highlighted in grey). Orange arrowheads indicate specific CLOuD9 construct target regions. The EcoRI fragment containing hypersensitivity sites 1–4 of the LCR (black bar) was used as the anchor region. Its crosslinking frequency with other indicated EcoRI fragments (names on the top of the graph) were assessed. The human β-globin genes and LCR hypersensitivity sites are depicted on the bottom of the graph with chromosomal position coordinates. Data from ChIP-seq of H3K4me3 and H3K9me3 demonstrate that this region is euchromatic in K562s. (**e**) Similar reversible changes in chromatin structure are seen in HEK 293T cells, despite evidence from H3K4me3 and H3K9me3 ChIP-seq data that the globin region is heterochromatic in this cell type. (**f**) 3C assay measuring Oct4 locus-wide crosslinking frequencies in 293T cells after 72 h of treatment with ABA (red) showing induced Oct4/distal enhancer contacts (highlighted in grey). Orange arrowheads indicate specific CLOuD9 construct target regions. The MboI fragment containing the Oct4 promoter (black bar) was used as the anchor region. Its crosslinking frequency with other indicated MboI fragments (names on the top of the graph) were assessed. The human Oct4 regions are depicted on the bottom of the graph with chromosomal position coordinates. All of the 3C results were obtained from at least three independent experiments. 3C values were normalized to tubulin. For β-globin, interaction frequencies between the anchor fragment and the fragment encompassing the β/HS fragment were set to zero. For Oct4, interaction frequencies between the anchor fragment and a negative control fragment outside of the Oct4 interacting region were set to zero. Error bars indicate s.d. *n*=3.

Our CLOuD9 technology consists of dCas9 proteins fused to a unique, reversible chemical induced proximity system that utilizes the plant phytohormone *S*-(+)-abscisic acid (ABA) and modified components of the plant ABA signalling pathway^11^ (Fig. 1a). Each of the dCas9 components are directed to a target locus using standard CRISPR guide RNAs (gRNAs), and juxtaposition of the two chromosomal loci is induced with addition of ABA which facilitates reversible association of the chemical induced proximity proteins (dimerization)^11^. Importantly, we made use of dCas9 from both *Staphylococcus aureus* (CSA) and *S. pyogenes* (CSP), tethering one half of the dimerization construct to the C terminus of each of the dCas9 proteins (Fig. 1b) and ensuring 100% functional juxtaposition of the two target genomic regions. In principle, this simple system offers the ease and flexibility of reversible chromosomal manipulation between any two genomic regions targetable by CRISPR gRNAs.

We chose to first test the utility of CLOuD9 on the human globin locus, given the well-characterized, dynamic chromosomal rearrangements that regulate globin gene expression during development (for review see ref. 1). As the K562 human erythroleukemia cell line has been exhaustively characterized to aberrantly express the fetal γ-globin gene at high levels, rather than the β-globin gene traditionally seen in adult human erythroid lineage cells, we chose to investigate whether we could re-establish β-globin gene expression in these cells. CLOuD9 constructs were targeted to the promoter of the β-globin locus (CSA) and the HS2 region of the LCR (CSP). Cells with CLOuD9 targeting either two separate β-globin promoter or two separate HS2 regions were used as controls. ABA was then added for varying durations to induce dimerization (Fig. 1c).

The correct localization and targeting of each CRISPR-dCas9 component was first confirmed by chromatin immunoprecipitation-quantitative PCR (ChIP-qPCR, Supplementary Fig. 1). In addition, to verify that CSA and CSP were indeed dimerizing upon addition of the ligand, co-immunoprecipitations were performed both in the presence and absence of ABA, as well as after ligand washout (Supplementary Fig. 2). As expected, addition of the ligand induced clear dimerization of CSA and CSP, and this effect was reversible upon ligand removal. We next sought to confirm that our forced dimerization induced a conformational change in the chromatin that was specific to the targeted sites. Indeed, as early as 24 h after the addition of ABA, an increase in the frequency of β-globin-LCR contacts was observed through chromosome conformation capture (3C) in cells containing both dimerization partners, but not in any of the controls (Fig. 1d and Supplementary Figs 3 and 4). Consistent with previous reports, we observed that induction of an LCR-β-globin contact did not destroy the endogenous LCR-globin contact—rather, the new LCR-β-globin contact appears in addition to the endogenous connection^10^. This effect was sustained throughout 72 h of dimerization regardless of the precise region within the LCR and β-globin promoter that was targeted (Supplementary Figs 5 and 6). Importantly, the chromatin loop modification induced by CLOuD9 was reversible, as 3C performed after ligand washout (in as little as 24 h) resulted in restoration of the LCR chromosomal configuration to its endogenous state (Fig. 1d and Supplementary Figs 3–6).

As the globin locus in K562s is a large span of euchromatin and thus may be more amenable to manipulation (Fig. 1d), we sought to determine whether loop formation would be possible in regions of heterochromatin. Accordingly, CLOuD9 constructs targeting the LCR and β-globin loci were introduced into HEK 293T cells, where the globin genes are not expressed and are heterochromatic (Fig. 1e). As observed in K562s, addition of ABA for 24 h resulted in an increase in the frequency of β-globin/LCR contacts as measured by 3C (Fig. 1e and Supplementary Fig. 3). This illustrates the robustness of CLOuD9 in its ability to operate in multiple cellular environments, regardless of endogenous chromatin state, accessibility or conformation.

As CLOuD9 is designed to be broadly applicable, we next sought to elucidate whether CLOuD9 could induce alterations in chromatin conformation at additional loci. We targeted CLOuD9 to the Oct4 promoter and distal 5′-enhancer in 293T cells where no endogenous contacts have been reported and Oct4 is not expressed at detectable levels. We chose to target these regions due to evidence that contact of the distal 5′-enhancer with the promoter of Oct4 drives expression of this gene in embryonic stem cells^12^. Similar to our observations at the β-globin locus, addition of ABA to CLOuD9-enabled cells resulted in induction of a contact between the Oct4 distal 5′-enhancer and promoter that was not present in control cells (Fig. 1f). Interestingly, induction of this contact also recruited a 3′-enhancer to the Oct4 promoter, consistent with previous reports that the 3′-enhancer is juxtaposed to the Oct4 promoter/5′distal enhancer complex during endogenous gene activation^13^.

### CLOuD9 induces context specific alterations at gene loci

**Figure 2:**
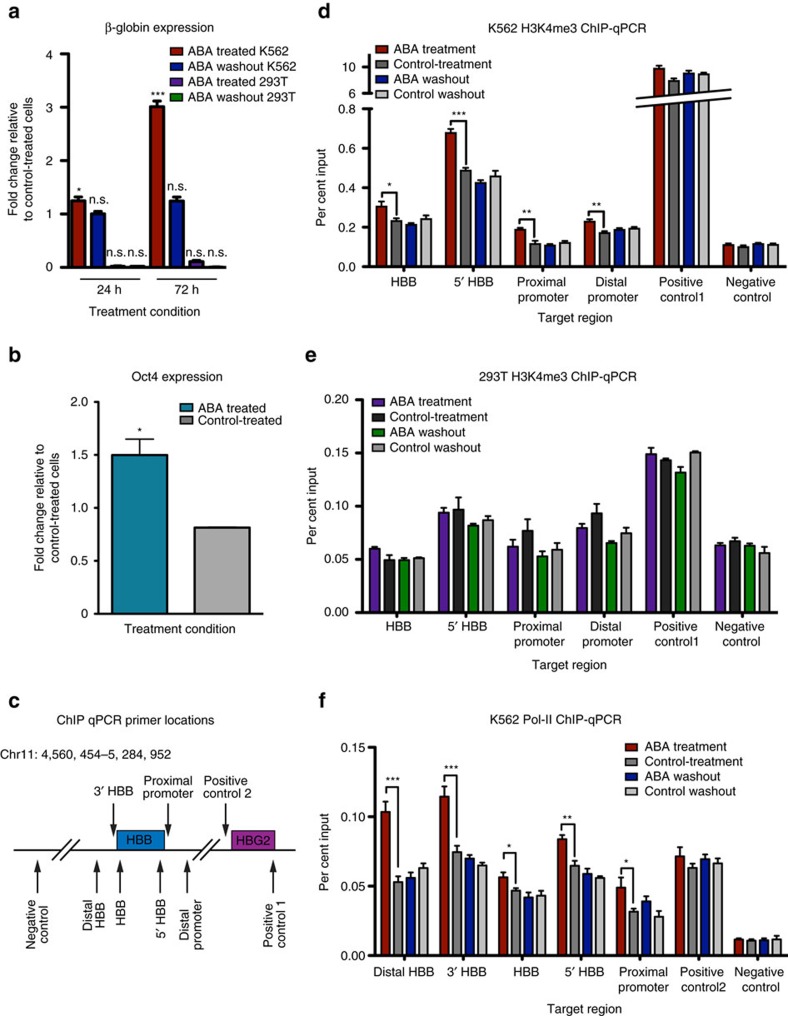
CLOuD9 induces context specific alterations in gene expression and chromatin state. (**a**) CLOuD9-induced chromatin looping at the β-globin locus results in reversible induction of β-globin expression in K562s but not in 293Ts. Significance given relative to control treated cells. Two-tailed student’s *t-*tests *P<0.05, *t*=3.418, df=5; ***P<0.0001, *t*=10.42 df=5; n.s. non-significant. Error bars indicate s.d. *n*=3. (**b**) Induction of Oct4 expression was observed in 293Ts following CLOuD9-induced looping at the same locus. Significance given relative to control treated cells. Two-tailed student’s *t-*tests *P<0.05, *t*=4.562, df=2. Error bars indicate s.d. (**c**) Schematic of ChIP-qPCR primer locations along the β-globin gene body. (**d**,**e**) ChIP-qPCR demonstrates reversible alterations in H3K4me3 at the β-globin locus in K562s but not in 293Ts following CLOuD9-induced looping. Two-tailed student’s *t-*tests *P<0.05, **P<0.001, ***P<0.0001. Error bars indicate s.d. (**f**) CLOuD9 mediated alterations in β-globin transcription in K562s correspond with increases in RNA Pol-II occupancy across the entirety of the β-globin gene body. Two-tailed student’s *t-*tests *P<0.05, **P<0.001, ***P<0.0001. Error bars indicate s.d.

We next investigated whether CLOuD9-induced chromatin conformation changes altered gene expression. As the LCR contact with the globin gene loci and the distal 5′-enhancer contact with the Oct4 promoter have been shown to be critical for transcription^1^,^13^, we reasoned that forcible loop induction would promote strong gene expression in both contexts. Indeed, qPCR with reverse transcription analyses demonstrated that for both loci, addition of ABA resulted in marked increases in gene expression compared to controls (Fig. 2a,b). Interestingly, while Oct4 levels were dramatically increased in 293Ts in response to dimerization at that locus, induction of a new LCR and β-globin contact in 293Ts and K562s significantly altered β-globin expression only in K562s (Fig. 2a). Addition of ABA for as little as 24 h drove significant changes in β-globin expression, and robust increases were observed following 72 h of ABA-induced dimerization (Fig. 2a). This effect was seen in ligand-treated cells containing dimerization partners directed to both the LCR and β-globin promoter simultaneously regardless of position (Supplementary Fig. 7), but was not observed in controls (Supplementary Fig. 8). Critically, endogenous levels of β-globin were restored upon ABA removal, regardless of whether initial dimerization was for 24 h or 72 h (Fig. 2a and Supplementary Fig. 7). In support of these observations, ChIP-qPCR of K562s and 293Ts showed increases in H3K4me3 along the β-globin gene only in K562s following dimerization, with baseline levels being restored following ligand washout (Fig. 2c–e). Furthermore, increased RNA Pol-II occupancy of the β-globin gene locus following dimerization corroborated the increased transcription of β-globin in K562s, an event that was also reversible after ligand removal^14^ (Fig. 2f). Taken together, these results highlight the importance of appropriate cellular context in driving functional consequences of loci juxtaposition.

### CLOuD9 establishes stable chromatin loops

**Figure 3:**
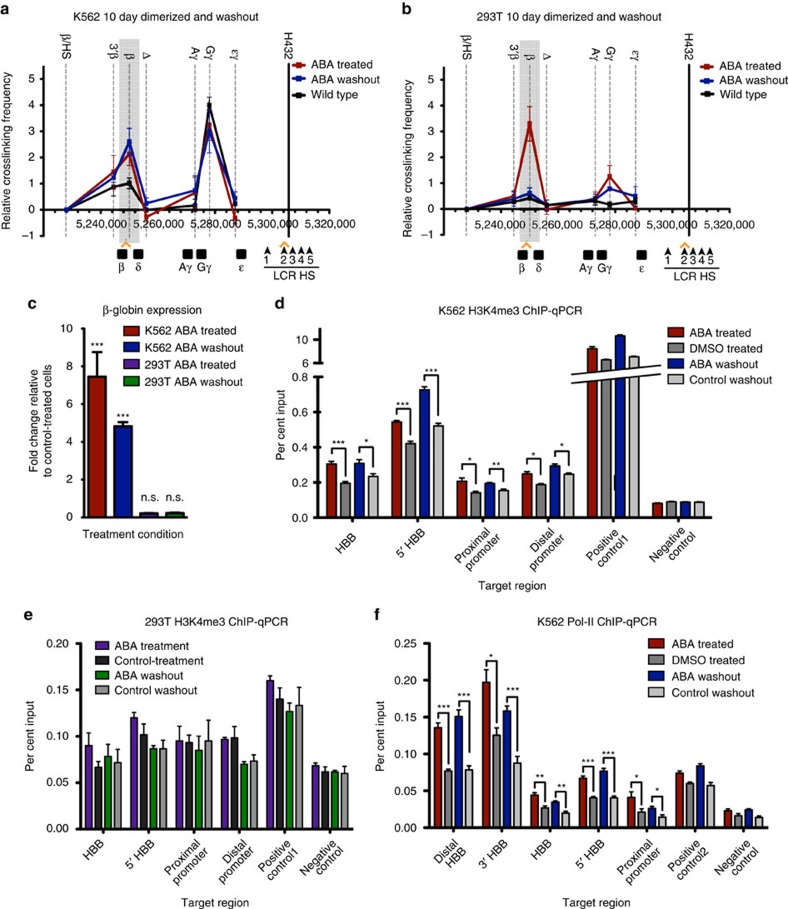
CLOuD9 establishes stable chromatin loops that sustain robust gene expression following long-term dimerization. (**a**,**b**) 3C assay demonstrates that in K562s but not 293Ts, CLOuD9-induced chromatin looping becomes irreversible after 10 days of ABA treatment, even when ABA is removed for up to 10 additional days. All 3C results were obtained from at least three independent experiments. 3C values were normalized to tubulin, and interaction frequencies between the anchor fragment and the fragment encompassing the β/HS fragment were set to zero. Error bars indicate s.d. *n*=3. (**c**) Loop stabilization in K562s results in persistent expression of β-globin, even following 10 days of ABA washout. No changes in β-globin expression are observed in 293Ts. Significance given relative to control treated cells. Two-tailed student’s *t-*tests ***P<0.0001, *t*=5.963, df=5; n.s. non-significant (**d**) ChIP-qPCR showing increases in H3K4me3 marks over the β-globin locus in response to CLOuD9-induced looping are sustained following 10 days of ligand washout in K562s. Two-tailed student’s *t-*tests *P<0.05, **P<0.001, ***P<0.0001. (**e**) No significant alterations in H3K4me3 signals following long-term dimerization were observed by ChIP-qPCR in 293Ts. (**f**) Increased RNA Pol-II occupancy of the β-globin locus following long-term loop induction was maintained in K562s following 10 days of ligand washout. Two-tailed student’s *t-*tests *P<0.05, **P<0.001, ***P<0.0001. All error bars indicate s.d.

Having established that CLOuD9 can reversibly manipulate chromatin contacts and induce corresponding alterations in gene expression, we next set out to investigate the impacts of long-term induced chromosomal looping. Following 10 days of ABA-induced dimerization, both K562 and 293T CLOuD9-transduced cells demonstrated increased β-globin-LCR contacts relative to controls, as observed previously (Fig. 3a,b and Supplementary Figs 9 and 10). As we had discovered previously, increased β-globin expression as a result of dimerization was only observed in K562s (Fig. 3c). Further, the location of the CLOuD9 construct within the β-globin promoter region had notable impacts on the strength of β-globin expression at this extended timepoint (Supplementary Fig. 7). Remarkably, regardless of construct location, removal of ABA in K562s, but not 293Ts, no longer resulted in the reversal of chromatin contacts (Fig. 3a,b and Supplementary Figs 9–11). Although a slight reduction in gene expression was observed following 10 days of ligand washout, a markedly elevated level of gene expression persisted (Fig. 3c). Consistent with these observations, ChIP-qPCR demonstrated significant increases in H3K4me3 relative to controls along the β-globin gene in K562s but not 293Ts (Fig. 3d,e). In addition, a significant increase in RNA Pol-II occupancy (relative to controls) along the same gene region was sustained following ligand washout in K562s as measured by ChIP-qPCR (Fig. 3f), suggesting stabilization of the induced chromatin contact driving sustained gene expression.

### RNA helicases DDX5 and DDX17 stabilize long-term chromatin loops

**Figure 4:**
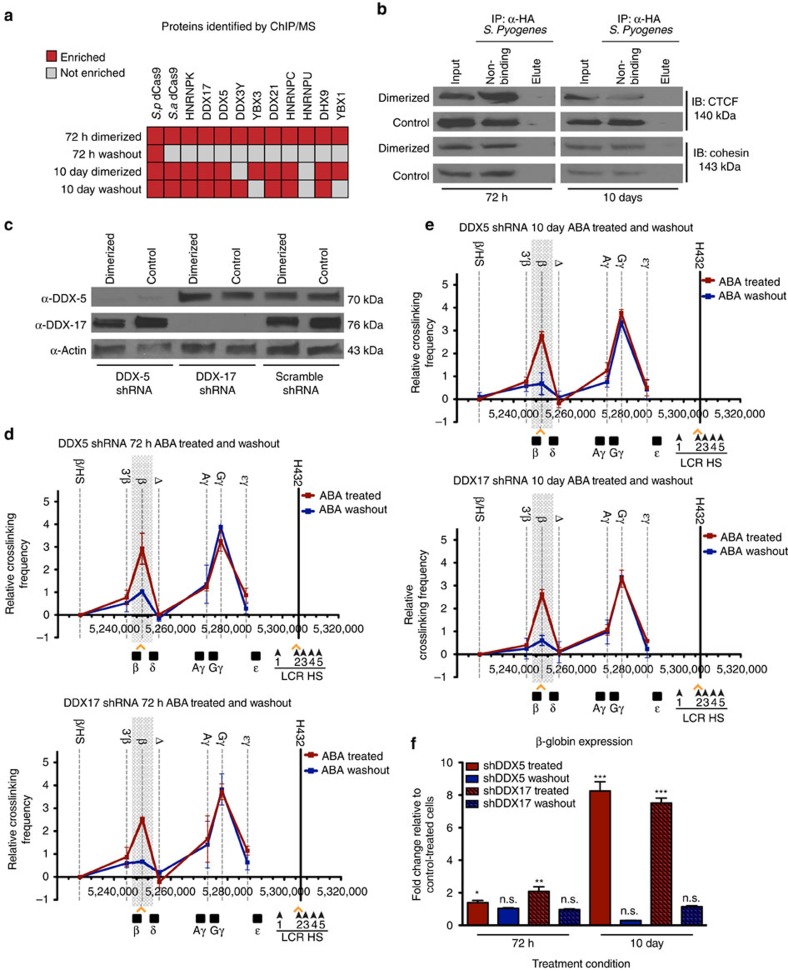
RNA helicases DDX5 and DDX17 stabilize long-term chromatin loops. (**a**) MS/ChIP of dimerized CLOuD9 cells after 72 h and 10 day treatments demonstrate differential enrichment of novel proteins at the induced looping loci after 10 days. (**b**) Immunoprecipitation of CLOuD9 complexes demonstrates that CTCF and cohesin were not found to be localized to the induced loops. (**c**) shRNA knockdown of DDX5 and DDX17 in K562s containing CLOuD9 constructs. (**d**,**e**) 3C demonstrates that while DDX5 and DDX17 knockdown do not affect induction of β-globin-LCR contacts, the induced chromatin loops are no longer stabilized following 10 days of dimerization and subsequent washout. 3C values were normalized to tubulin, and interaction frequencies between the anchor fragment and the fragment encompassing the β/HS fragment were set to zero. Error bars indicate s.d. *n*=3. (**f**) Endogenous β-globin expression is restored following short- and long-term dimerization and subsequent ligand washout in DDX5 and DDX17 knockdown cells. Significance given relative to control treated cells. Two-tailed student’s *t*-tests *P<0.05, *t*=2.538, df=6; **P<0.001, *t*=3.791, df=6; ***P<0.0001, shDDX5 *t*=12.6, df=6, shDDX17 *t*=19.35, df=6; n.s. non-significant. All error bars indicate s.d.

To determine the mechanisms underlying preservation of the chromatin conformation with long-term induced dimerization, we performed mass spectrometry analysis following ChIP (MS/ChIP) on CLOuD9 cells dimerized for 72 h and 10 days. As shown in Fig. 4a, after 72 h of induced looping, the CLOuD9-associated dCas9 proteins, a small number of RNA helicases, and members of the heterogeneous nuclear ribonucleoprotein machinery are preferentially enriched in the dimerized samples. However, longer duration chromatin loop formation was accompanied by recruitment of similar RNA helicases and heterogeneous nuclear ribonucleoproteins to the contact region, that remained at the site of induced contact even after 10 subsequent days of ligand washout (Fig. 4a and Supplementary Data 1), implying a functional role for these proteins in maintaining the early stages of *de novo* chromatin contacts. Notably absent from the MS analyses are the traditional regulators of chromatin architecture, CCCTC-binding factor (CTCF) and cohesin (Fig. 4a,b; Supplementary Fig. 12 and Supplementary Data 1), suggesting a possible novel regulatory mechanism of RNA helicases in stabilizing genome topology. Recruitment of the most robust RNA helicases in the MS/ChIP samples, DDX5 and DDX17, which we found to co-immunoprecipitate (Supplementary Fig 13), was confirmed by ChIP-qPCR (Supplementary Fig 14). To test the possible involvement of these RNA helicases in the long-term maintenance of *de novo* chromatin contacts, we performed shRNA-mediated knockdown of both DDX5 and DDX17 (Fig. 4c and Supplementary Fig. 15). Stable knockdowns were made only of either protein individually, as simultaneous knockdown of both proteins resulted in cell death. Interestingly, while knockdown of either protein had no impact on loop formation or gene expression following 72 h or 10 days of ABA-mediated β-globin/LCR dimerization (Fig. 4d–f and Supplementary Figs 16 and 17), long-term dimerization followed by ligand washout no longer resulted in sustained chromatin looping, and β-globin expression following ligand washout returned to baseline levels (Fig. 4e,f and Supplementary Fig. 17).

## Discussion

Taken together, we have built CLOuD9, to the best of our knowledge a new, broadly applicable tool for the precise manipulation of three-dimensional chromatin structure. Using this tool, we demonstrate that chromatin looping alone is sufficient to alter gene expression in the proper biological context. In addition, we have identified a novel mechanism for stable formation of *de novo* chromatin contacts independent of cohesin and CTCF, through the RNA helicases DDX5 and DDX17. Whether these or other RNA helicases are broadly involved in maintaining *de novo* chromatin contacts will be an important area of future study.

The findings reported here indicate that CLOuD9 can be utilized to elucidate more precisely how chromatin structure regulates gene expression and enhance our understanding of the role of large-scale chromatin organization in the control of transcriptional dynamics. This will be particularly important in contexts such as cancer and congenital disorders, where disruptions of genomic organization by chromosomal rearrangements markedly affect gene expression^15^,^16^,^17^,^18^. Understanding how chromatin restructuring may be harnessed in these contexts to regulate gene expression for therapeutic benefit will be of significant importance in future studies. Taken together, future work with CLOuD9 technology will undoubtedly shed greater light on the hierarchy and dynamics of chromatin domains that facilitate chromatin restructuring, as well as how both *de novo* and sustained loops can be harnessed and reorganized to alter transcriptional programs in development and disease.

## Methods

### Cell culture

Wild-type K562 cells, a gift from Dr Ravindra Majeti, were cultured in RPMI 1640 media (Life Technologies, 11875–119) with addition of 10% fetal bovine serum (FBS) and 1% penicillin/streptomycin. Cells were maintained in 25 cm^2^ canted neck flasks, and were adjusted to a density of 400,000 cells per ml daily. Following transduction with CLOuD-9 constructs, cells were maintained in media supplemented with 2 μg ml^−1^ puromycin and 100 μg ml^−1^ hygromycin.

Wild-type 293T cells, a gift from Dr Howard Chang, were cultured in DMEM media (Life Technologies, 11995-065) with addition of 10% FBS and 1% penicillin/streptomycin. Cells were maintained in 10 cm^2^ plates and passaged when confluent. Following transduction with CLOuD-9 constructs, cells were maintained in media supplemented with 1 μg ml^−1^ puromycin and 25 μg ml^−1^ hygromycin.

### Development of CLOuD-9 plasmids

Briefly, beginning with lentiCRISPR v2 (Addgene plasmid #52961), the gRNA and Cas-9 sequences were replaced with either *S. aureus* nuclease-deficient Cas-9 and compatible gRNA sequence (CSA) or *S. pyogenes* nuclease deficient Cas-9 and compatible gRNA sequence (CSP). The reversibly dimerizeable ABI1 and PYL1 domains were then added to the modified Cas-9 sequences. The gRNA sequences utilized are in Supplementary Table 1.

### Lentivirus production

Lentivirus was produced using sequence verified packaging constructs pRSV-Rev (Addgene plasmid #12253), pMD2.G (Addgene plasmid #12259), and pMDLg/pRRE (Addgene plasmid #12251). Briefly, 750,000 293T cells per well were seeded into a six-well plate. Twenty-four hours after seeding, media was changed to fresh antibiotic-free DMEM (Life Technologies, 11995-065) with 10% FBS. Plasmids were transfected into 293Ts using Lipofectamine 2000 following manufacturer’s protocol (Thermo Fisher Scientific 11668). After 12 h, media was changed to viral production media (RPMI 1640 for K562s, DMEM for 293Ts, both with 10% FBS). Forty-eight hours later, viral production media was collected and spun down at low speed to remove any cell debris. Virus was then used immediately for transduction or frozen at −80 °C for future use.

### Lentivirus transduction

K562s were transduced through addition of 250 μl of each viral construct of interest to a 15 ml conical tube containing 80,000 cells. Total volume of media in each conical was brought to 1 ml with antibiotic-free RPMI 1640+10% FBS, and polybrene was added to each tube to a final concentration of 4 μg ml^−1^. Cells were then spun at 800*g* for 30 min at room temperature, resuspended briefly by pipetting without removing the viral supernatant, and moved to cell culture plates. Twenty-four hours later, cells were spun down at 300*g* for 5 min at room temperature, viral supernatant was aspirated, and cells were resuspended and re-plated in RPMI 1640+10% FBS+1% penicillin/streptomycin overnight. On the following day, puromycin (2 μg ml^−1^) and hygromycin (100 μg ml^−1^) were added to the culture medium to select for transduced cells.

293Ts were transduced through addition of 250 μl each viral construct of interest to one well of a six-well plate. Total volume of media was brought to 2 ml with antibiotic-free DMEM+10% FBS, and polybrene was added to a final concentration of 2 μg ml^−1^. Twenty-four hours later, viral supernatant was aspirated, and media was changed to DMEM+10% FBS+1% penicillin/streptomycin overnight. On the following day, puromycin (1 μg ml^−1^) and hygromycin (25 μg ml^−1^) were added to the culture medium to select for transduced cells.

For both cell lines, cells were kept in selection media for at least 1-week before use in any downstream experiments, and were maintained in selection media for the duration of all experiments.

### Cell dimerization and washout

For dimerization treatment of CLOuD-9-transduced cells, 1 mM Abscisic Acid (or an equivalent volume of DMSO for controls) was added to the culture medium. Abscisic acid was used within 6 months of date of receipt, and was kept cold, protected from light throughout use. Media was changed and fresh abscisic acid or DMSO was added daily.

For washout and reversal of dimerization, cells that had been subjected to dimerization were spun down, washed once in PBS, and resuspended in fresh culture medium without dimerization agent.

### Immunoprecipitation and co-immunoprecipitations

Cells dimerized as described above were collected and spun down before crosslinking with 1% formaldehyde at room temperature for 10 min followed by glycine quenching. Cells were lysed in 0.1 M Tris pH 7.5, 10 mM potassium acetate, 15 mM magnesium acetate, 1% NP-40 and spun to isolate nuclei, then nuclei were lysed using 0.1 M Tris pH 8, 1% SDS, 10 mM EDTA. Nuclei were sonicated briefly to solubilize material, and SDS was quenched with dilution buffer containing 0.01% SDS, 1.1% Triton X-100, 1.2 mM EDTA, 16.7 mM Tris pH 7.5 and 167 mM NaCl. Protein complexes were immunoprecipitated overnight using the antibodies against HA (Cell Signaling 3724), Flag (Sigma F1804), or DDX5 (Bethyl A300-523A), all at 1:50, and were washed three times with 100 mM Tris pH 9, 100 mM LiCl, 1% NP-40, and 1% sodium deoxycholate. Complexes were eluted by vortexing twice with 1% SDS, 15 mM NaHCO_3_ for 15 min each time. Elutes were run on SDS-page gels and probed with antibodies against the HA tag (Cell Signaling 3724), Flag tag (Sigma F1804), CTCF (Cell Signaling 2899), SMC1 (Bethyl A300-055A), or DDX17 (Bethyl A300-509A) as indicated, all at 1:1,000.

### RNA extraction and quantitative PCR

Total RNA was isolated using TRIzol (Life Technologies 15596-018) and RNeasy Kit (QIAGEN 74106) according to the manufacturer’s protocol. cDNA was made with Superscript VILO (Life Technologies 11754-050). All primers utilized were previously reported^10^, but are also summarized in Supplementary Table 1. qPCR analyses were performed using SYBR Green I MasterMix (Roche 4707516001) on the Light Cycler 480II (Roche).

### Statistical analysis of gene expression changes

Statistical analysis was performed using GraphPad Prism 5 for MacOS X. For every sample, two sets of duplicates were averaged for each of three biological replicates to obtain a final n of three for all statistical analyses. Two-tailed student’s *t*-tests were performed on ABA and control treated samples. Error bars represent the standard deviation.

### Chromosome conformation capture assay

3C assays of the β-globin locus were performed as previously described^10^, with the following modifications. Cells were crosslinked with 1% formaldehyde at room temperature for 10 min followed by glycine quenching, cell lysis, EcoRI digestion and T4 ligation. 3C ligation products were quantified in two sets of duplicates for each of three biological replicates by quantitative SYBR Green real-time PCR using SYBR Green I MasterMix (Roche 4707516001) on the Light Cycler 480II (Roche). The HS432 fragment was used as the anchor fragment for all experiments. Samples were normalized to 3C signals from the tubulin locus. To eliminate variability between samples, interaction frequencies between the anchor fragment and the fragment encompassing the β/HS fragment were set to zero. Primer sequences are listed in Supplementary Table 1.

3C assays of the Oct4 locus were also performed as previously described^13^, with the following modifications. Cells were crosslinked with 1% formaldehyde at room temperature for 10 min followed by glycine quenching, cell lysis, MboI digestion and T4 ligation. 3C ligation products were quantified in two sets of duplicates for each of three biological replicates by PCR amplification and Image J quantification of amplicon intensity. Primer sequences are listed in Supplementary Table 1.

### Mass spectrometry chromatin immunoprecipitation

Immunoprecipitations were performed as described above. Briefly, cells were crosslinked with 1% formaldehyde at room temperature for 10 min followed by glycine quenching, cell lysis, nuclei isolation and lysis, and brief sonication. Protein complexes were immunoprecipitated overnight using an anti-HA antibody (Cell Signaling 3724), and were washed and eluted as described above. Elutes were used directly for mass spectrometry experiments without further dilution.

### CLOuD-9 mass spectrometry sample preparation

Samples were reduced and alkylated by diluting 2 × the sample’s volume with exchange buffer containing 8 M urea (Acros Organics, New Jersey) and 100 mM ammonium bicarbonate (Sigma-Aldrich, St Louis MO). Dithioteitol (DTT; Sigma-Aldrich, St Louis MO) was added to a final concentration of 10 mM DTT. Samples were incubated at room temperature for 1.5 h. Iodoacetamide (Acros Organics, New Jersey) was added in 1.5-fold molar excess of DTT followed by another incubation for 1 h at room temperature in the dark. Samples were buffer exchanged using a filtered aided sample preparation method^12^. First, samples were transferred into a Microcon Ultrafiltration 10 kDa filter (ED Millipore, Billerica MA, Cat#MRCPRT010) and centrifuged at 14,000*g* for 30 min. Two-hundred microlitre of exchange buffer was added and the 30 min centrifugation and addition of exchange buffer was repeated three times. Next, 200 μl of digestion buffer (50 mM ammonium bicarbonate) was added to the Microcon filter followed by centrifugation at 14,000*g* for 25 min and repeated three additional times. Samples were digested with 6 μg of trypsin (Promega, Sunnyvale, CA) at 37 °C with shaking at 900 r.p.m. for 18 h. After digestion, samples were centrifuged at 14,000*g* for 15 min to collect tryptic peptides. Peptides were dried using a SpeedVac (Labconco, Kansas City, MO). Samples were further desalted by C18 Zip-Tips (EMD Millipore, Darmstadt, Germany, Cat#ZTC18S096).

### LC/MS analysis

Tryptic peptides were loaded onto a C18 nanospray column (50 mm length, 2 μm particle size, Thermo Fisher Scientific, San Jose, CA) and separated by reversed-phase chromatography using a nanoEasy nLC-1200 (Thermo Fisher Scientific, San Jose, CA). Eluted peptides were analysed subject to online liquid chromatography–mass spectrometry analysis using a Q Exactive mass spectrometer (Thermo Fisher Scientific, San Jose, CA.). Mobile phase A consisted of 0.1% formic acid in water and the LC was pumped at 250 nl min^−1^. Mobile phase B (0.1% formic acid in acetonitrile) was run at 2% for the first 5 min, slowly ramped up to 20% in 150 min, and rapidly increased to 95% in 40 min. The top 15 most abundant ions per MS1 scan were selected for higher energy collision induced dissociation (27 eV) in a data-dependent fashion. MS1 resolution was set at 70,000, AGC target was set to 3e6, and the *m/z* scan range was set from *m/z*=375–1,500. MS2 resolution was set at 17,500 and AGC target at 2e5. Dynamic exclusion was enabled for 30 s.

### CLOuD-9 ChIP-qPCR

Conventional ChIP-qPCR was performed. Briefly, cells were crosslinked with 1% formaldehyde at room temperature for 10 min followed by glycine quenching, cell lysis, nuclei isolation and lysis and sonication to obtain 150–200 bp DNA fragments. Complexes were immunoprecipitated overnight using 10 μg of anti-HA tag (Cell Signaling 3724), anti-Flag tag (Sigma F1804), anti-H3K4me3 (AbCam ab8580), anti-RNA Pol-II (Active Motif 61083) or anti-DDX5 (Bethyl A300-523A). Real-time qPCR of purified DNA was performed using SYBR Green I MasterMix (Roche 4707516001) on the Light Cycler 480II (Roche). qPCR primers are provided in Supplementary Table 1.

### shRNA Knockdown of DDX5 and DDX17

MISSION shRNA bacterial stocks (TRCN0000272488, TRCN0000287046) were obtained from Sigma-Aldrich, and prepared as recommended by manufacturer. shRNA plasmids were isolated using a Qiagen Miniprep kit (Qiagen 27106) and lentiviral constructs were produced and delivered to K562 cells as described above. Efficient knockdown was validated by western blot.

### Protein extraction and western blot analysis

Cellular extracts were prepared using lysis buffer containing 50 mM Tris HCL (pH 7.5), 250 mM NaCl, 1% NP-40, 0.5% Na-deoxycholate, 0.1% SDS, and EDTA free protease inhibitor (Roche 11873580001). Extracts were run on a 4–12% Tris-Glycine gel (BioRad) and transferred onto PVDF membranes. Blots were blocked in 5% milk PBS-T for 1 h at room temperature followed by overnight incubation at 4 °C with primary antibodies at 1:1,000 (anti-HA Tag, Cell Signaling 3724S; anti-Flag Tag, Sigma F1804; anti-DDX5, Bethyl A300-523A; anti-DDX17, Bethyl A300-509A). Horseradish peroxidase (HRP) -conjugated secondary antibodies were used at 1:10,000 (anti-rabbit HRP, Santa Cruz sc-2030) or 1:1,000 (anti-mouse HRP, Cell Signaling 7076S).

### ChIP-seq sample acquisition and analysis

K562 and HEK293 ChIP-Seq data were obtained from Encode (ENCSR000AKU, ENCSR000APE, ENCSR000FCJ) and GEO (GSM1479215). Alignment was performed using Bowtie, and duplicates were removed using samtools. The filtered files were converted to bigwig format for visualization using genomeCoverageBed from Bedtools, as well as bedGraphToBigWig. The bigwig files were normalized using bamCoverage.

### Data availability

K562 and HEK293 ChIP-Seq data were obtained from Encode (ENCSR000AKU, ENCSR000APE, ENCSR000FCJ) and GEO (GSM1479215). All relevant data from this manuscript is available from the authors.

## Additional information

**How to cite this article:** Morgan, S. L. *et al*. Manipulation of nuclear architecture through CRISPR-mediated chromosomal looping. *Nat. Commun.* **8,** 15993 doi: 10.1038/ncomms15993 (2017).

**Publisher’s note**: Springer Nature remains neutral with regard to jurisdictional claims in published maps and institutional affiliations.

## Supplementary Material

Supplementary Information

Supplementary Data 1
