# Supplementary material for: Manipulation of nuclear architecture through CRISPR-mediated chromosomal looping
Source: Nat Commun. 2017 Jul 13;8:15993. doi: 10.1038/ncomms15993 (PMC5511349; doi:10.1038/ncomms15993)
Supplement: Supplementary Information [file ncomms15993-s1.pdf]

File name: Supplementary Information
Description: Supplementary figures and supplementary table.

File name: Supplementary Data 1
Description: Mass spectrometry peptides. Raw peptide identifications are provided for each sample. Each tab represents an individual mass spectrometry run, and is labeled with the sample name, treatment condition, and treatment duration.

Supplementary Fig. 1

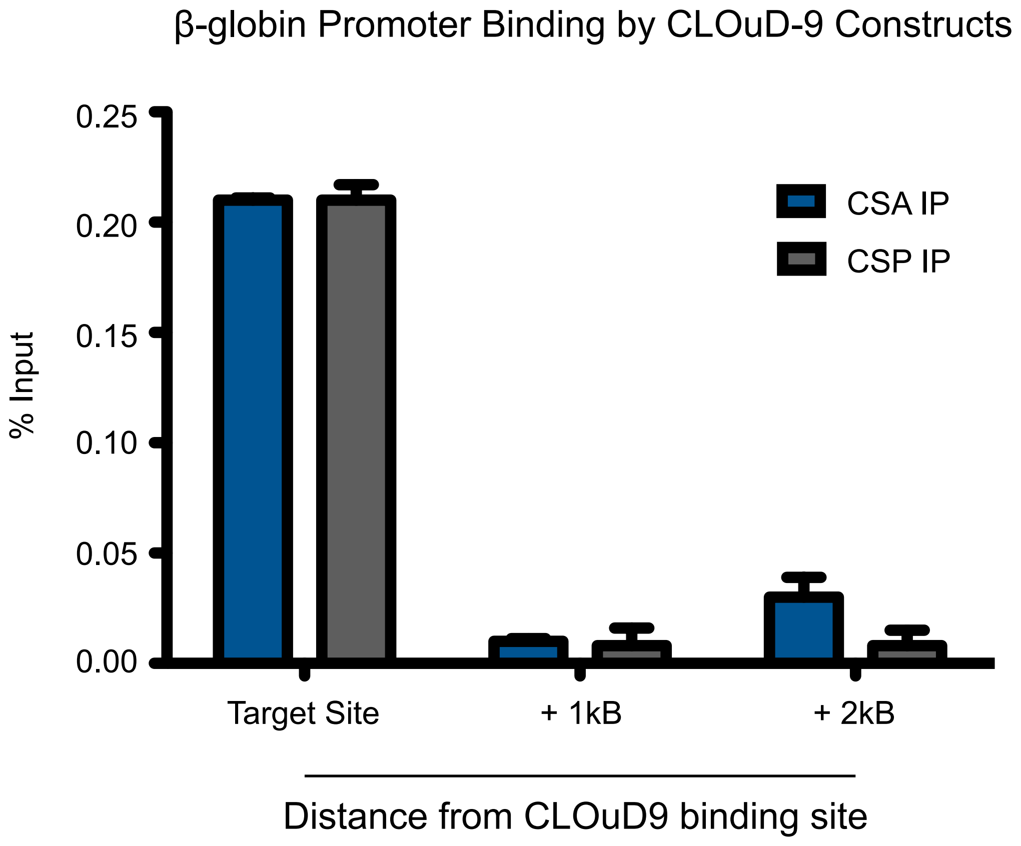

Supplementary Figure 1. CLOuD9 constructs localize to their intended target regions. Chromatin immunoprecipitation and quantitative PCR of CLOuD9 constructs demonstrates correct localization to their intended genomic loci.

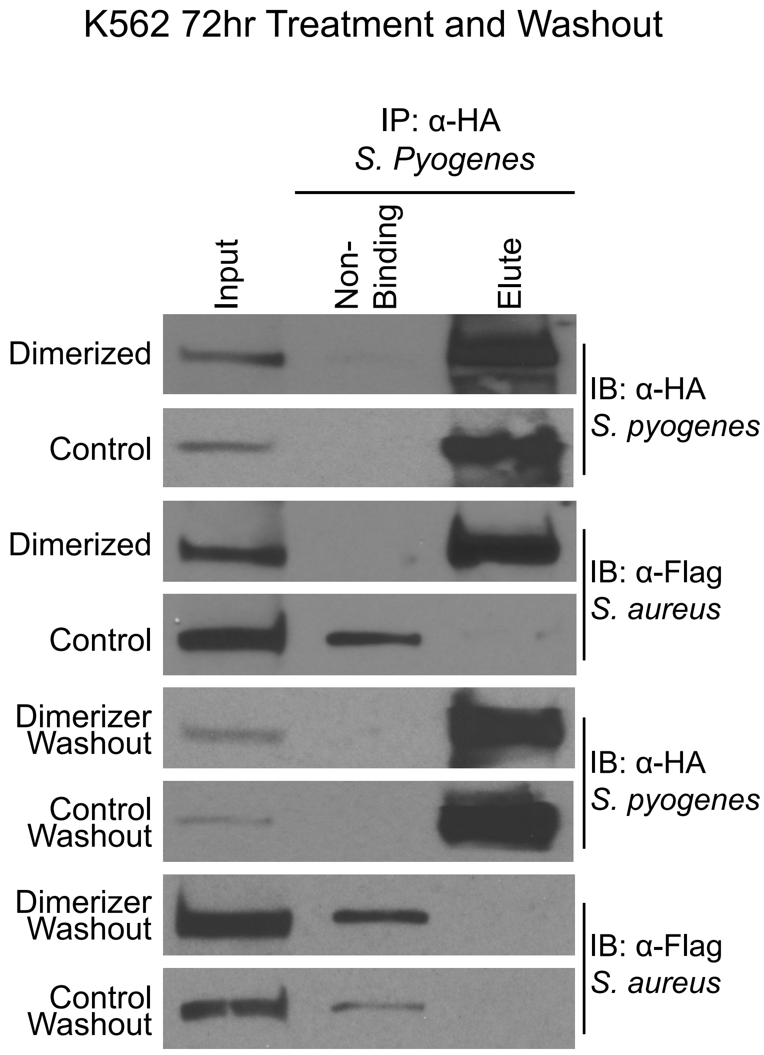
K562 72hr Treatment and Washout
IP: α-HA
S. Pyogenes
Input
Non-
Binding
Elute
Dimerized
Control
IB: α-HA
S. pyogenes
Dimerized
Control
IB: α-Flag
S. aureus
Dimerizer
Washout
Control
Washout
IB: α-HA
S. pyogenes
Dimerizer
Washout
Control
Washout
IB: α-Flag
S. aureus

Supplementary Figure 2. CLOuD9 constructs reversibly associate in response to ABA treatment. Co-immunoprecipitations demonstrating association of the dCas9 proteins following 72 hours of ABA treatment is reversed following subsequent 72 hours of ligand washout.

Supplementary Fig. 3

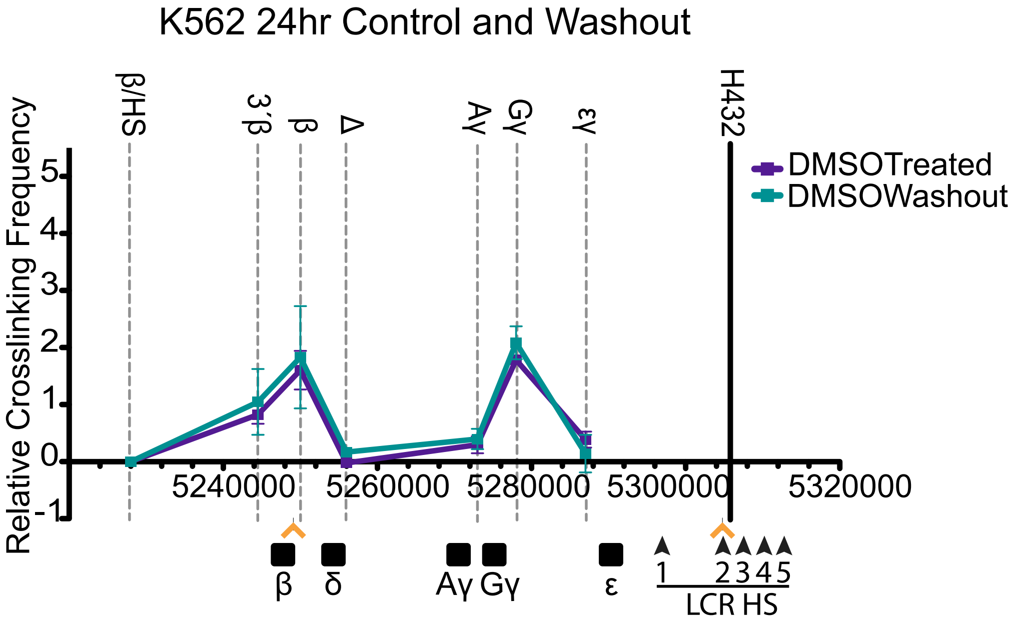

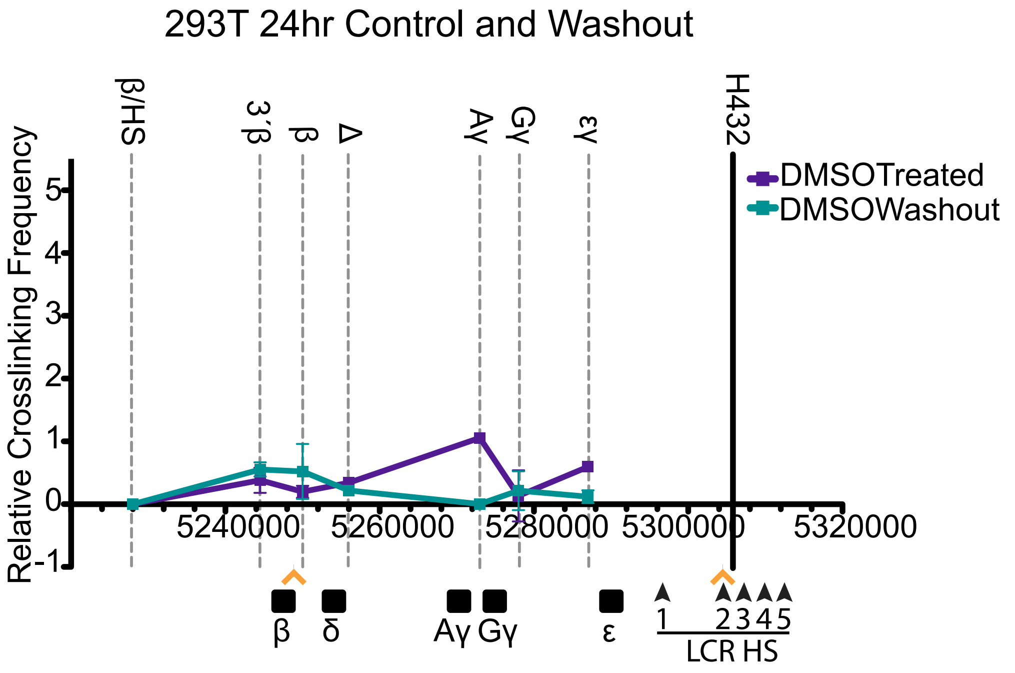

Supplementary Figure 3. Control treatment induces no changes in chromatin contacts. Treatment with DMSO, a control agent, for 24 hours induces no changes in the endogenous chromatin conformation by 3C in either K562 cells or HEK 293Ts. 3C values were normalized to tubulin, and interaction frequencies between the anchor fragment and the fragment encompassing the β/HS fragment were set to zero. Error bars indicate SD. n = 3.

Supplementary Fig. 4

LCR Targeting Constructs

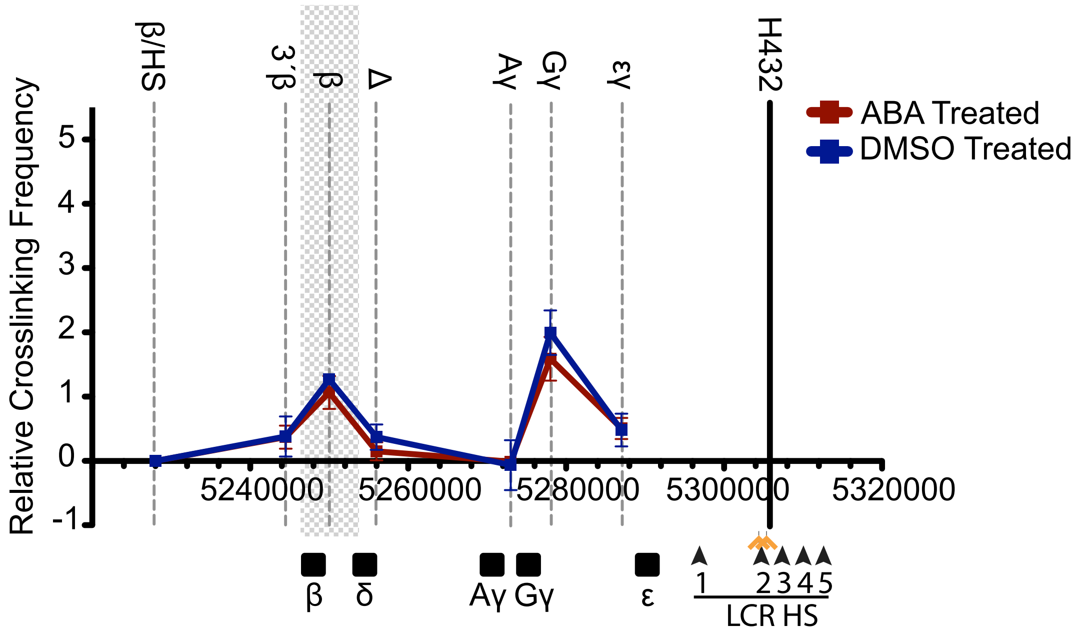

β-Globin Promoter Targeting Constructs

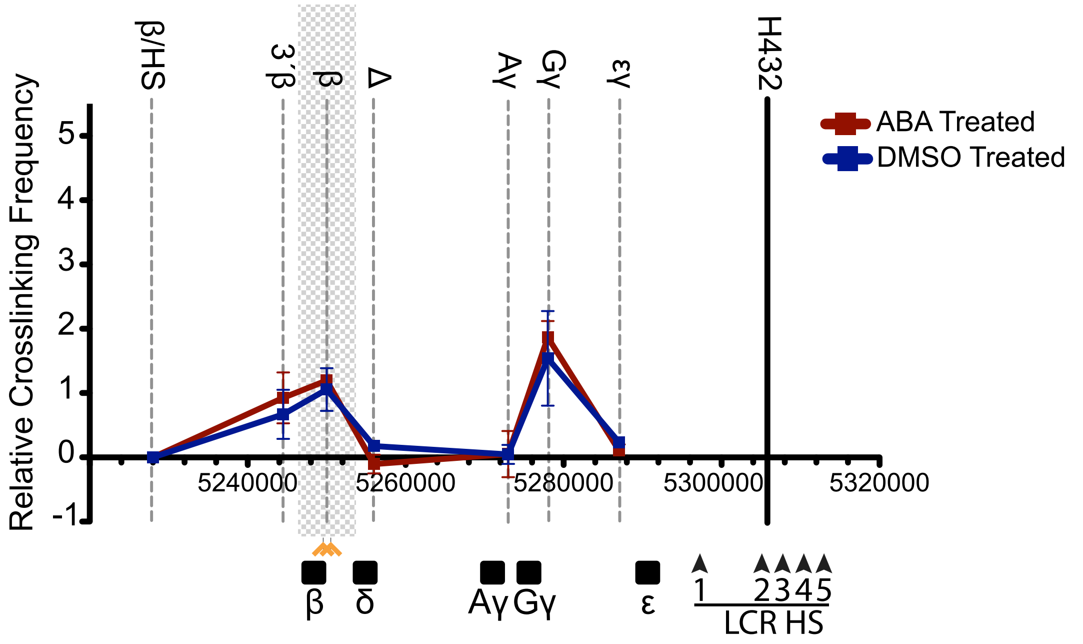

Supplementary Figure 4. Control CLOuD9 transduced cells show no alterations in chromatin looping. Directing two CLOuD9 constructs to either the LCR or the β-globin promoter induces no significant changes in chromatin structure by 3C following ABA treatment relative to control treatment. 3C values were normalized to tubulin, and interaction frequencies between the anchor fragment and the fragment encompassing the β/HS fragment were set to zero. Error bars indicate SD. n = 3.

Supplementary Fig. 5

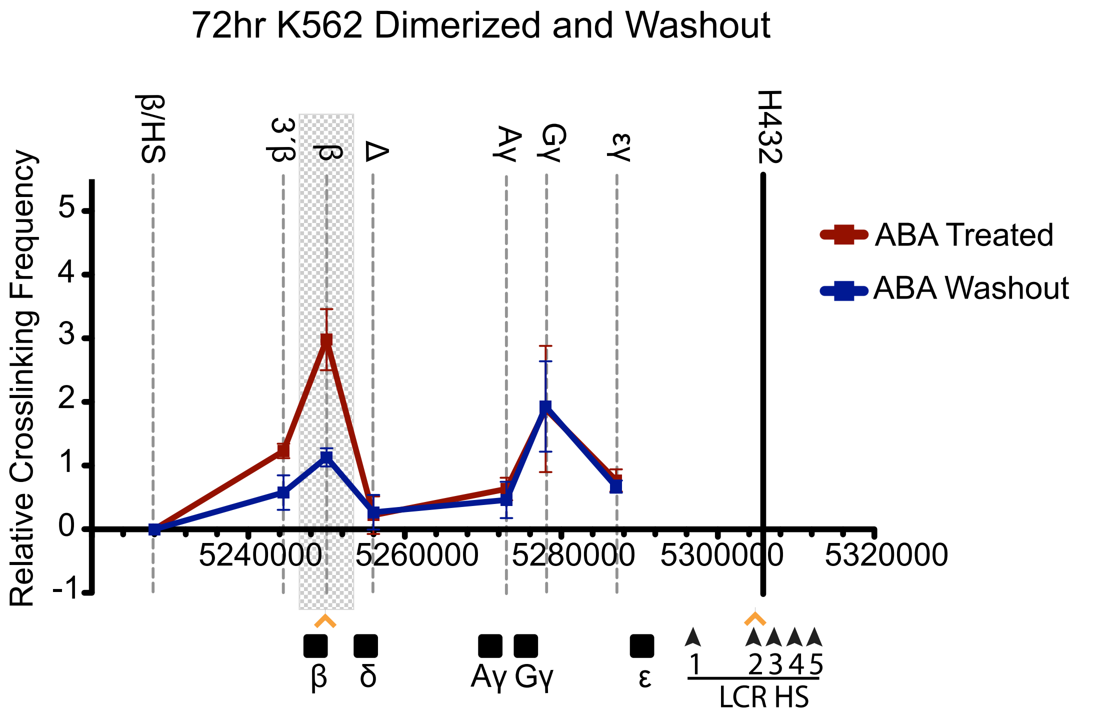

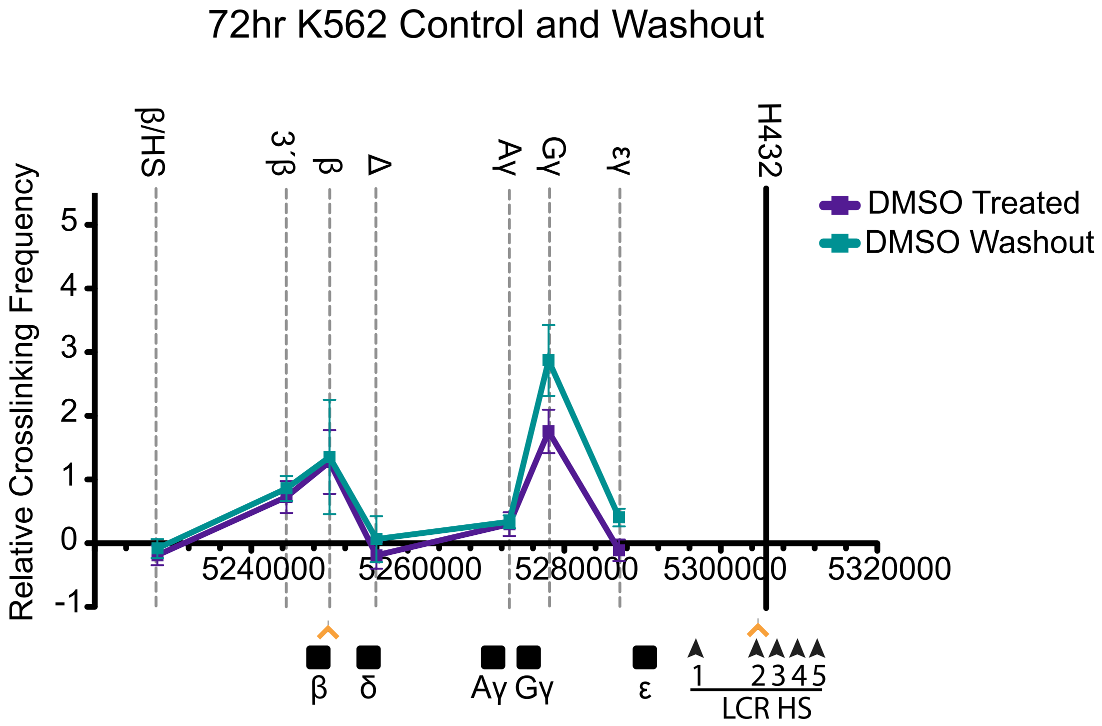

Supplementary Figure 5. CLOuD9 chromatin looping remains reversible after 72 hours of dimerization. 3C assay in K562s demonstrates reversibility of CLOuD9 induced β-globin/LCR contacts after 72 hours of ABA treatment. 3C values were normalized to tubulin, and interaction frequencies between the anchor fragment and the fragment encompassing the β/HS fragment were set to zero. Error bars indicate SD. n = 3.

Supplementary Fig. 6

K562 Guide Pair 2 - 72hr ABA Treated and Washout

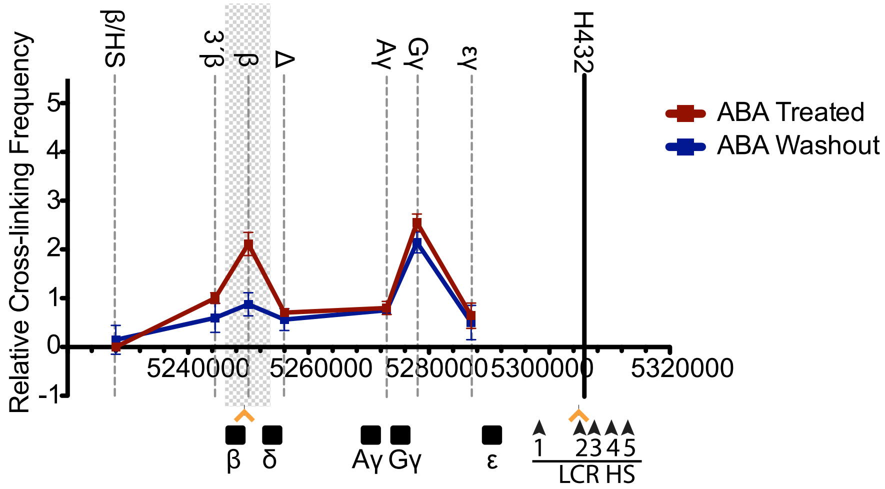

K562 Guide Pair 2 - 72hr DMSO Treated and Washout

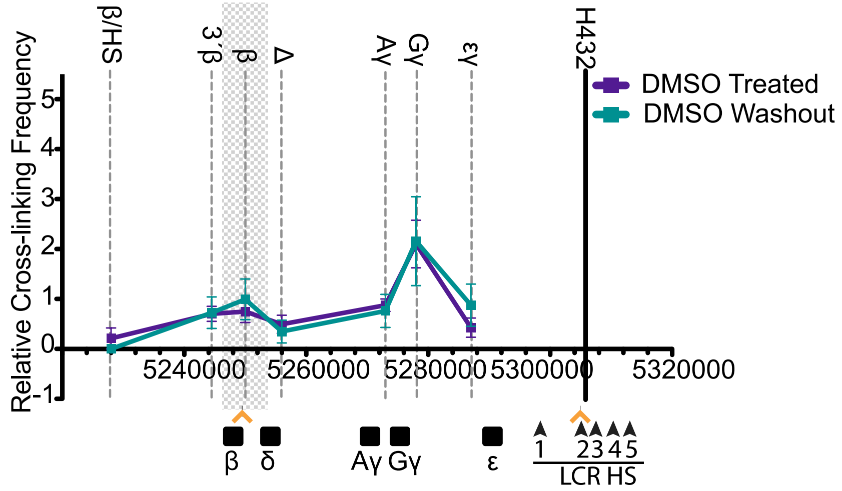

Supplementary Figure 6. CLOuD9 induced β-globin/LCR looping is not impacted by globin target site. Directing CSA and CSP constructs to alternate regions of the LCR or the β-globin promoter results in similar reversible changes in loop induction by 3C following 72 hours of ABA treatment. 3C values were normalized to tubulin, and interaction frequencies between the anchor fragment and the fragment encompassing the β/HS fragment were set to zero. Error bars indicate SD. n = 3.

Supplementary Fig. 7

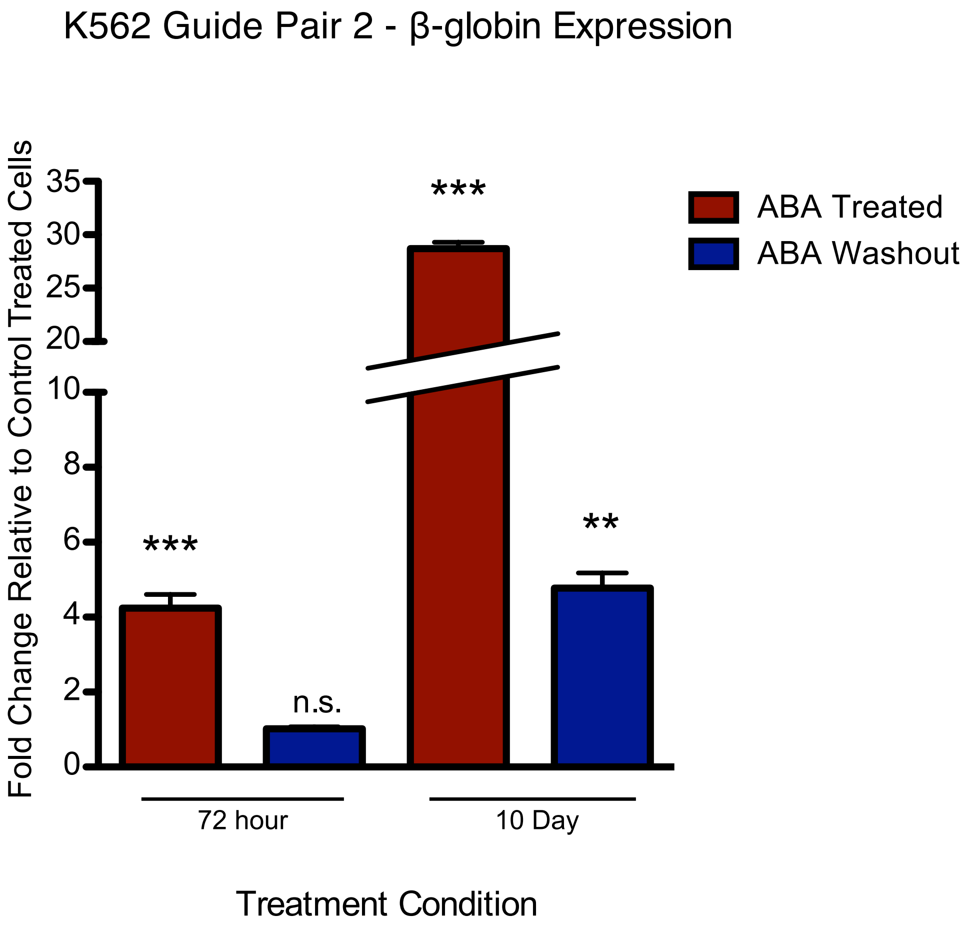

Supplementary Figure 7. CLOuD9 induced alterations in gene expression are sustained regardless of globin target site. Directing CSA and CSP constructs to alternate regions of the β-globin promoter and LCR has no impact on induction of gene expression following 72 hours of dimerization. However, while some impact on the strength of gene expression following long-term (10 day) dimerization was observed, high levels of β-globin relative to control treated cells were sustained following subsequent ligand washout for 10 additional days. Significance given relative to control treated cells. **$p < 0.001$, t = 10.25, df = 5; ***$p < 0.0001$, left to right t = 8.697, df = 6, t = 40.31, df = 7; n.s. non-significant. All error bars indicate SD.

Supplementary Fig. 8

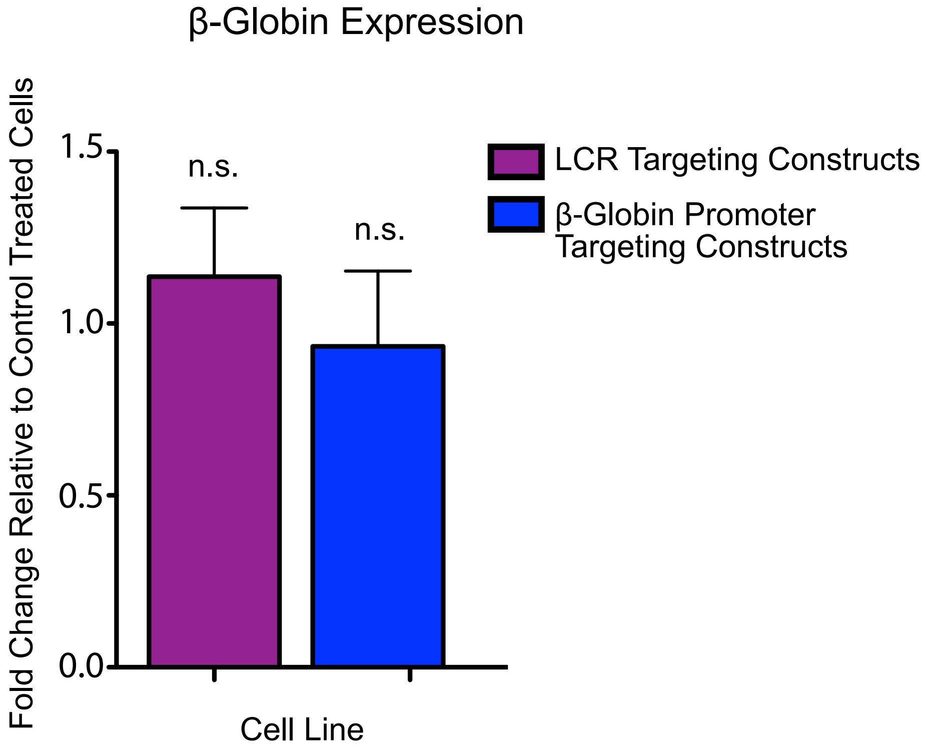

Supplementary Figure 8. Control CLOuD9 transduced cells show no alterations in β-globin expression. Directing two CLOuD9 constructs to either the LCR or the β-globin promoter induces no significant changes in β-globin expression following ABA treatment relative to control treatment. Significance given relative to control treated cells. n.s. non-significant.

Supplementary Fig. 9

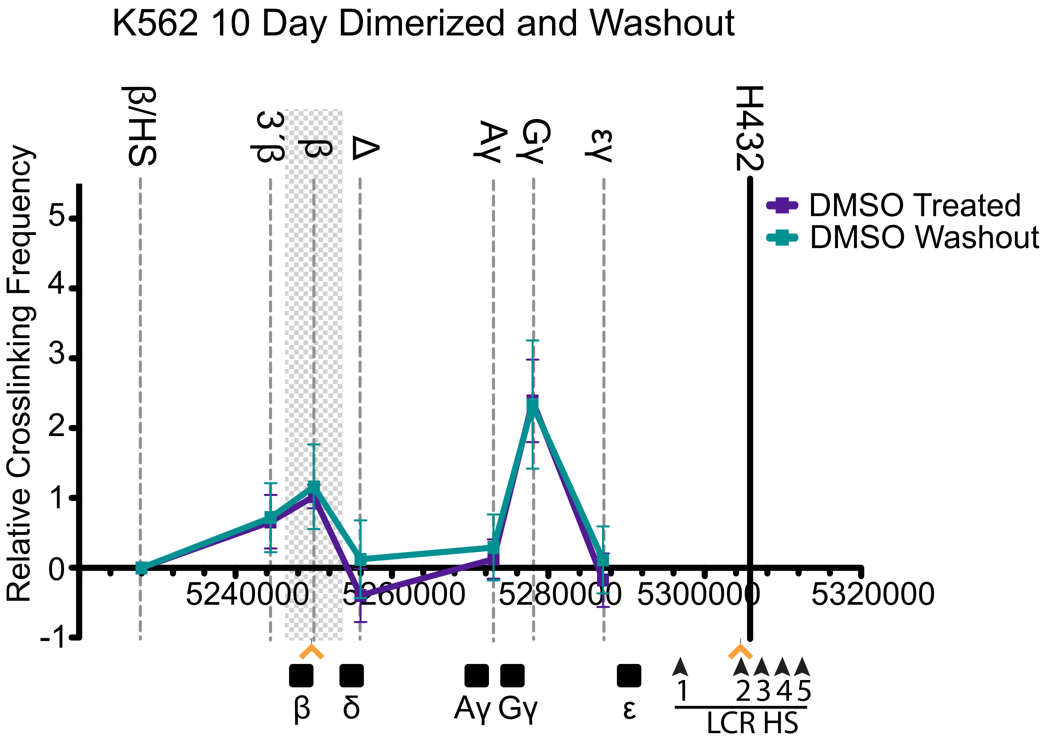

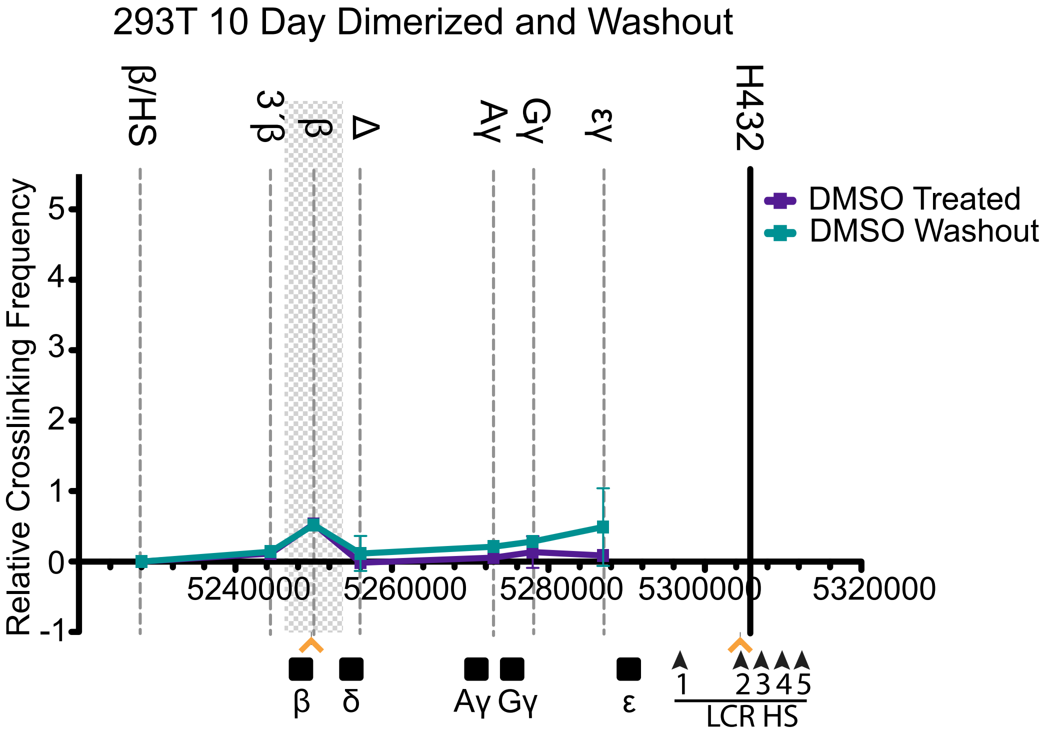

Supplementary Figure 9. Long-term control treatment induces no changes in chromatin contacts. Treatment with DMSO, a control agent, for 10 days induces no change in endogenous chromatin conformation by 3C in either K562 cells or HEK 293Ts. 3C values were normalized to tubulin, and interaction frequencies between the anchor fragment and the fragment encompassing the β/HS fragment were set to zero. Error bars indicate SD. n = 3.

Supplementary Fig. 10

K562 Guide Pair 2 - 10 Day ABA Treated and Washout

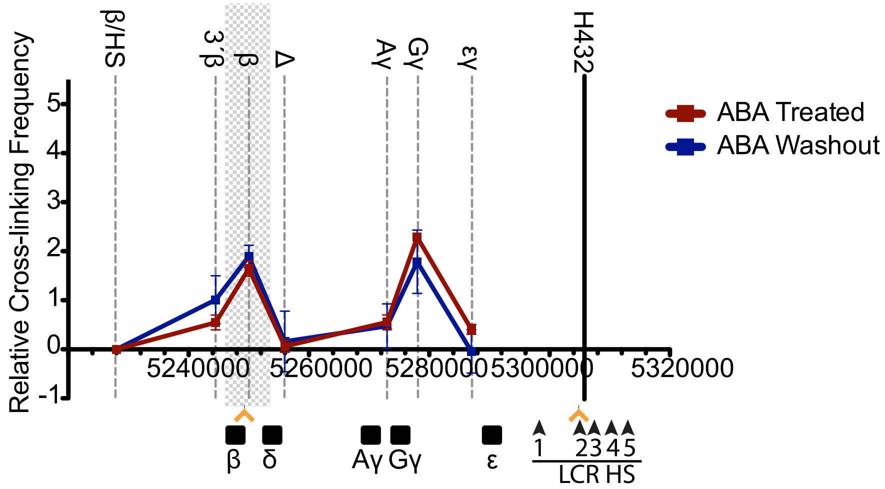

K562 Guide Pair 2 - 10 Day DMSO Treated and Washout

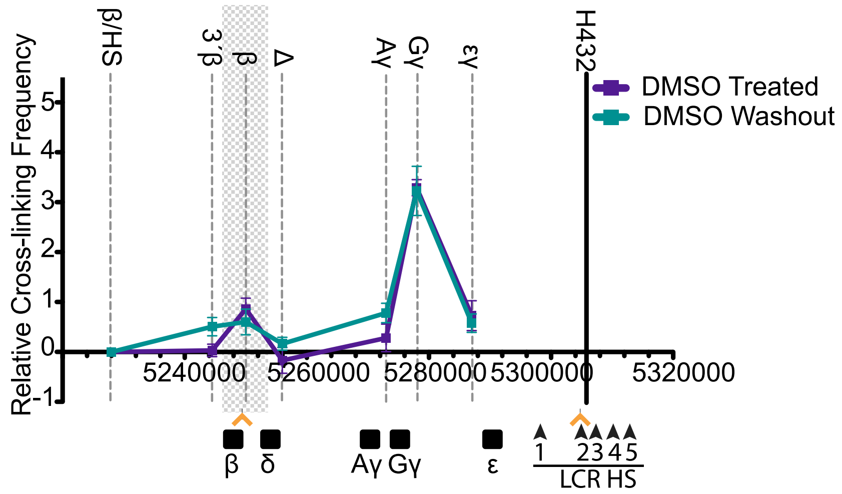

Supplementary Figure 10. Long-term CLOuD9 induced β-globin/LCR looping is not impacted by globin target site. Directing CSA and CSP constructs to alternate regions of the LCR or the β-globin promoter results in similarly sustained loop induction as demonstrated by 3C following 10 days of ABA treatment and 10 days of subsequent ligand washout. 3C values were normalized to tubulin, and interaction frequencies between the anchor fragment and the fragment encompassing the β/HS fragment were set to zero. Error bars indicate SD. n = 3.

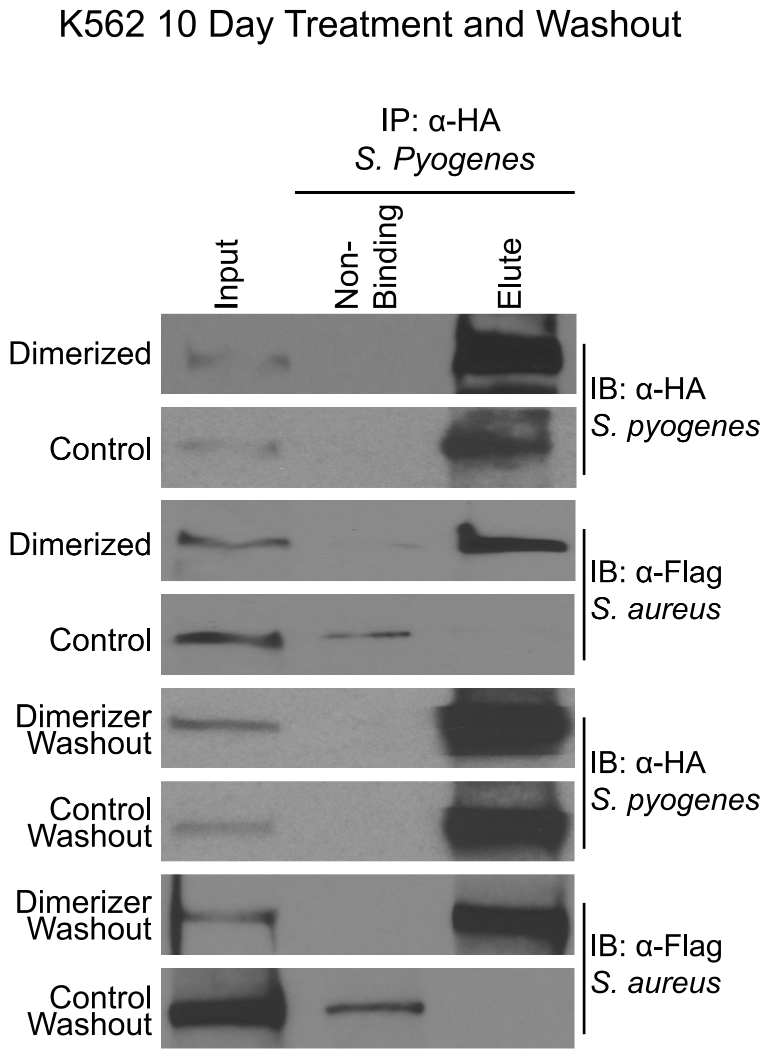
K562 10 Day Treatment and Washout
IP: α-HA
S. Pyogenes
Input
Non-Binding
Elute
Dimerized
Control
Dimerized
Control
Dimerizer Washout
Control Washout
Dimerizer Washout
Control Washout
IB: α-HA *S. pyogenes*
IB: α-Flag *S. aureus*
IB: α-HA *S. pyogenes*
IB: α-Flag *S. aureus*

Supplementary Figure 11. CLOuD9 constructs irreversibly associate in response to long-term ABA treatment. Co-immunoprecipitations demonstrating irreversible association of the CSA and CSP dCas9 proteins following 10 days of ABA treatment and 10 subsequent days of ligand washout.

Supplementary Fig. 12

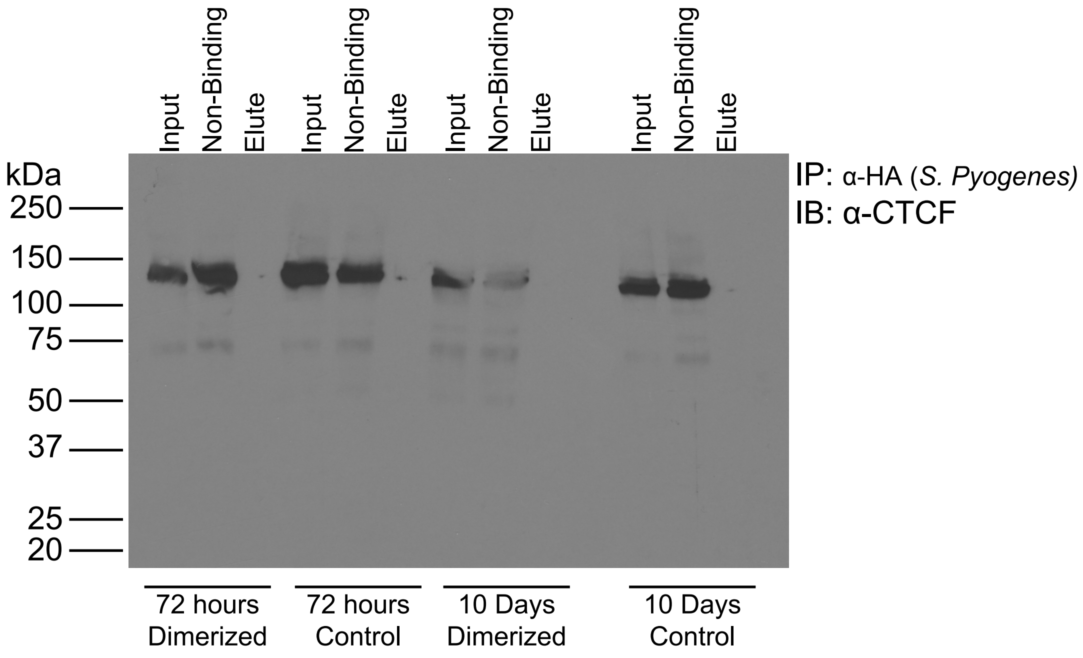

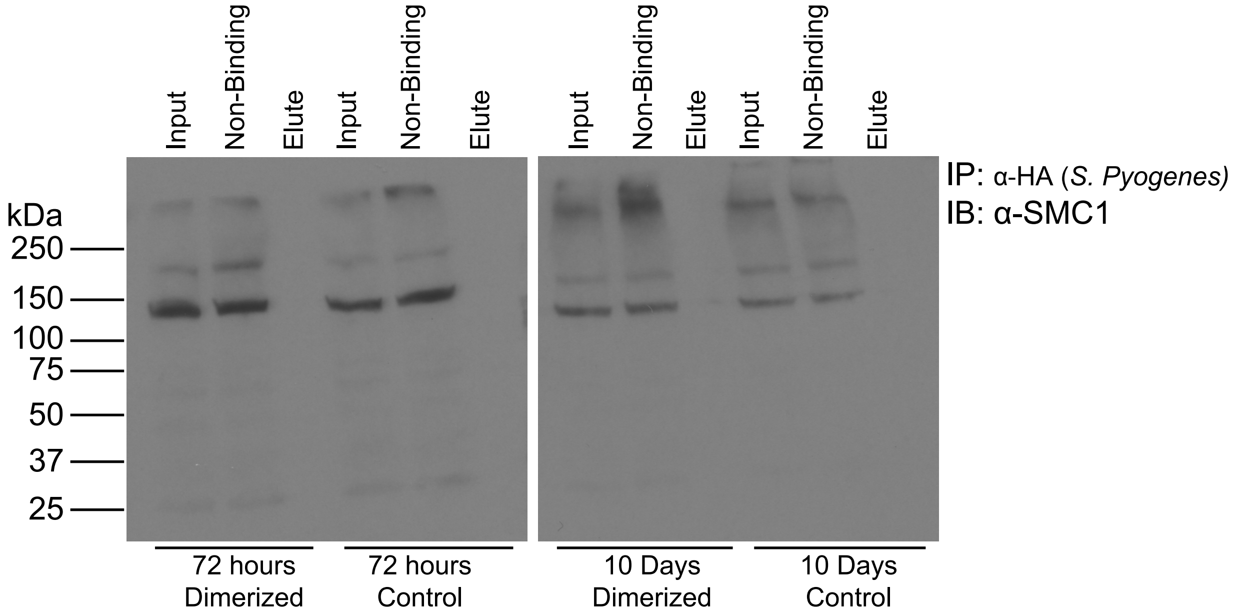

Supplementary Figure 12. Uncropped images of immunoblots in Fig 4b.
Immunoprecipitation of CLOuD9 complexes demonstrates that CTCF and cohesin were not found to be localized to the induced loops.

Supplementary Fig. 13

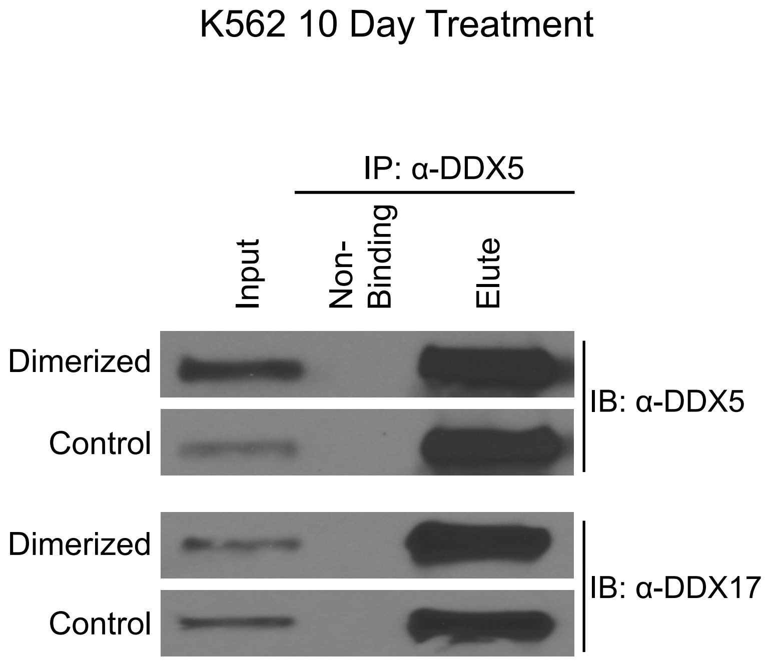

Supplementary Figure 13. RNA helicases DDX5 and DDX17 co-associate. Co-immunoprecipitations demonstrating DDX17 associates with DDX5 in K562 cells regardless of treatment condition.

Supplementary Fig. 14

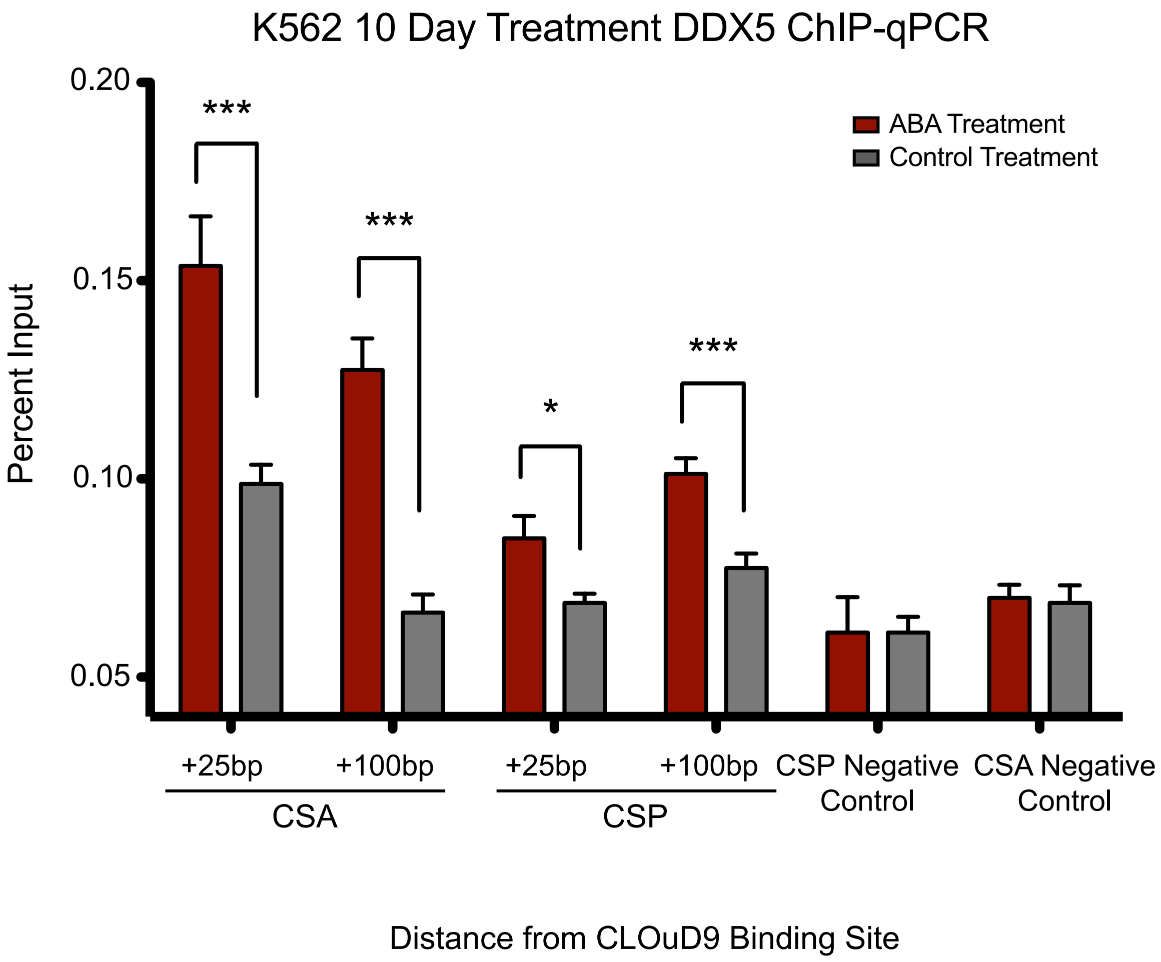

Supplementary Figure 14. RNA helicases DDX5 and DDX17 localize to CLOuD9 target regions. Chromatin immunoprecipitation and quantitative PCR of CLOuD9 constructs demonstrates their localization to regions of chromatin immediately adjacent to CLOuD9 target sites. *$p < 0.05$, t = 2.662, df = 14; ***$p < 0.0001$, left to right t = 4.1, df = 14, t = 6.659, df = 14; t = 4.392, df = 14. All error bars indicate SD.

Supplementary Fig. 15

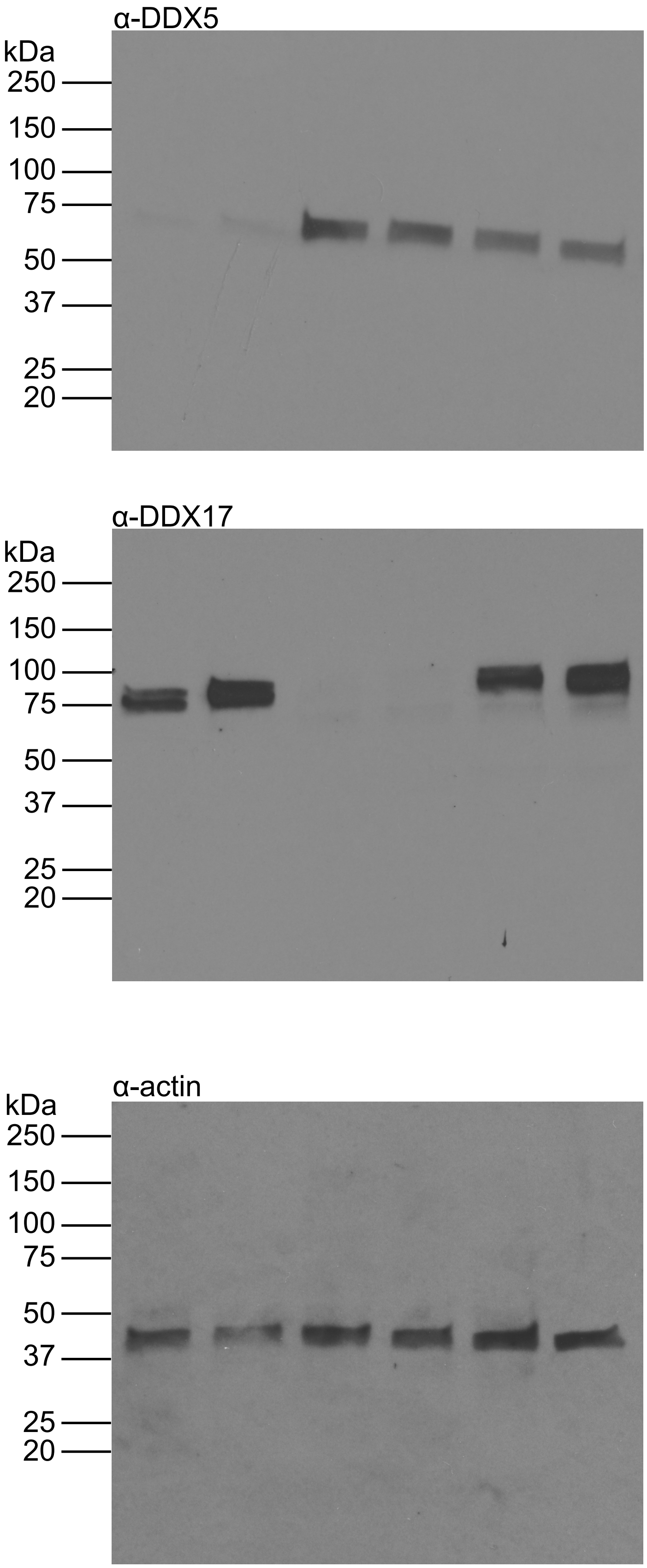

Supplementary Figure 15. Uncropped images of immunoblots in Fig 4c. shRNA knockdown of DDX5 and DDX17 in K562s containing CLOuD9 constructs.

Supplementary Fig. 16

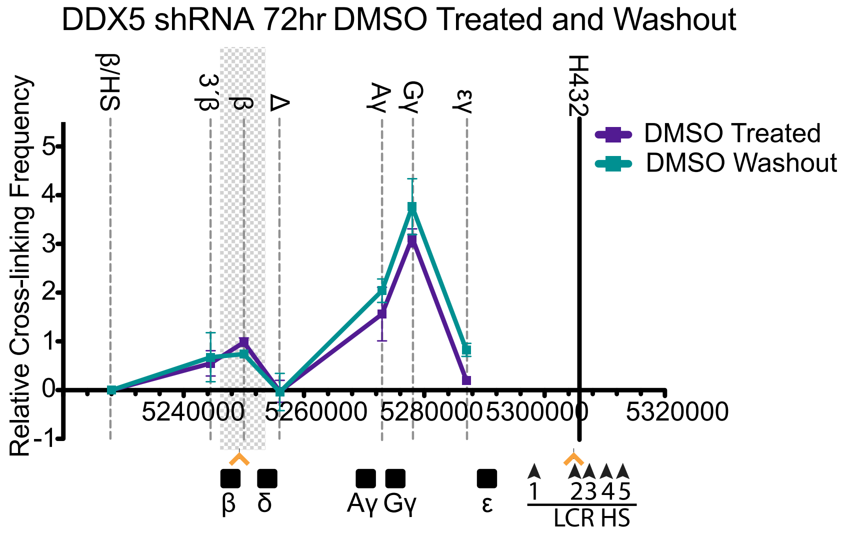

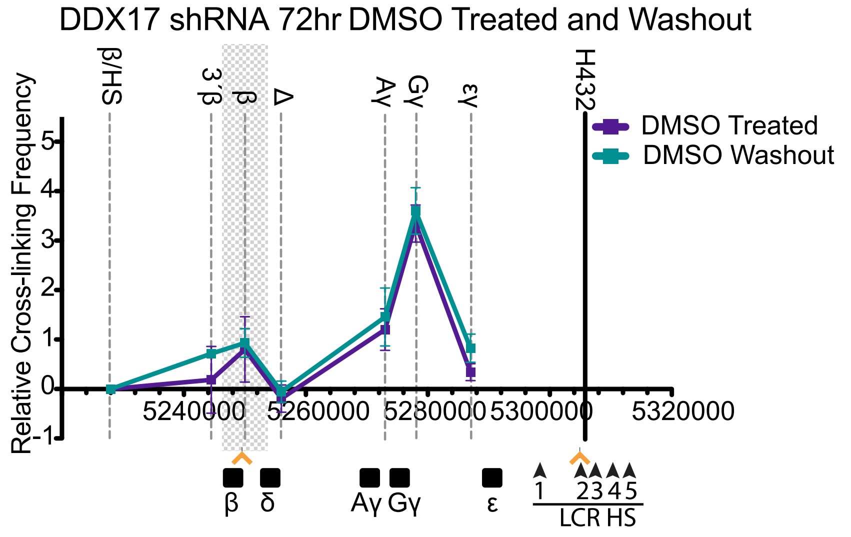

Supplementary Figure 16. Control treatment of DDX5 and DDX17 knockdown CLOuD9 cells induces no changes in chromatin contacts. Treatment with DMSO, a control agent, for 72 hours induces no changes in endogenous chromatin conformation by 3C in DDX5 or DDX17 knockdown K562 cells. 3C values were normalized to tubulin, and interaction frequencies between the anchor fragment and the fragment encompassing the β/HS fragment were set to zero. Error bars indicate SD. n = 3.

Supplementary Fig. 17

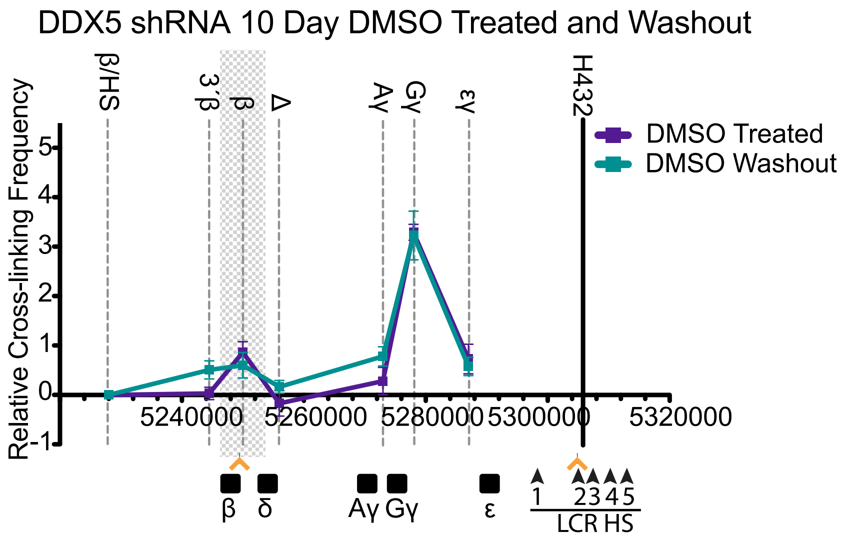

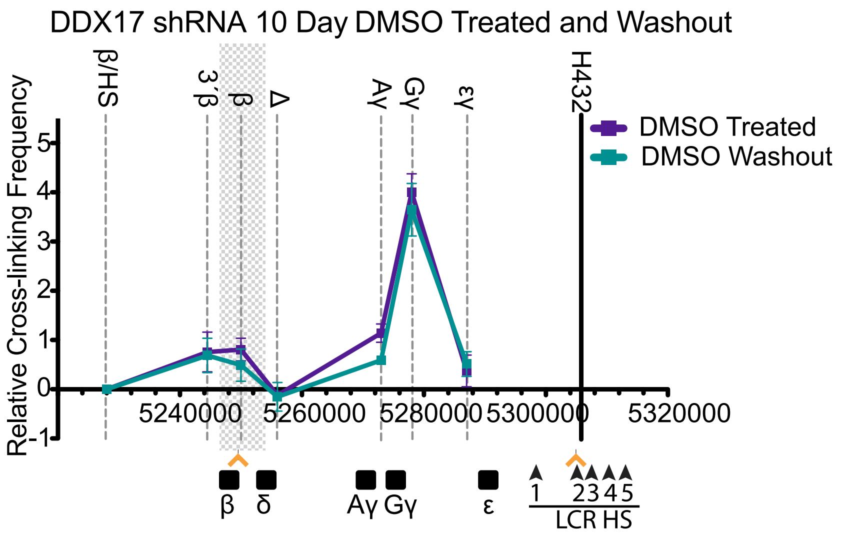

Supplementary Figure 17. Long-term control treatment of DDX5 and DDX17 knockdown CLOuD9 cells induces no changes in chromatin contacts. Treatment with DMSO, a control agent, for 10 days induces no changes in endogenous chromatin conformation by 3C in DDX5 or DDX17 knockdown K562 cells. 3C values were normalized to tubulin, and interaction frequencies between the anchor fragment and the fragment encompassing the β/HS fragment were set to zero. Error bars indicate SD. n = 3.

Supplementary Table 1. List of primer sequences for gRNAs, qRT-PCR, 3C, and ChIP qPCR.

**gRNA Sequences**		
Name	Sequence 5'-3'	CLOuD9 Construct
HBB Promoter Pair 1	TAGTCTGGGTATACTTAGAGG	CSA
LCR Pair 1	CTAGAGTGATGACTCCTATC	CSP
HBB Promoter Pair 2	AAGTTGATGCACTAAAAGTGG	CSA
LCR Pair 2	AATATGTCACATTCTGTCTC	CSP
Oct4 Promoter	CTTATGGCTGTTGATGCATTG	CSA
Oct4 Distal 5'Enhancer	CTCTTTGGATCGCGTCACTC	CSP
**qPCR Primers**		
Primer	Forward 5'-3'	Reverse 5'-3'
β-globin	TGGGCAACCCTAAGGTGAAG	GTGAGCCAGGCCATCACTAAA
Oct4	TGTACTCCTCGGTCCCTTTC	TCCAGGTTTTCTTTCCCTAGC
GapDH	ACCACAGTCCATGCCATCACT	CCATCACGCCACAGTTTCC

**3C Primers**	
β-globin	
Name	Primer Sequence 5'-3'
3C B/HS	TCTTAGAAAGCCTTTACAATTTCCTTTATC
3C 3 Beta	AGCTTAGTGATACTTGTGGGCCA
3C Beta	GCTCGGCACATGTCCCATCCAG
3C Delta	AAAAAATGTGGAATTAGACCCAGGAATG
3C 5 Delta	GGGTGTGTATTTGTCTGCCA
3C G/A	AATTTGAAGATACAGCTTGCCTCCGATAAG
3C Gg	GGGTTCATCTTTATTGTCTCCT
3C E/G	CCACCCCGATAAAGATTTTTCTCCATCA
3C HS432	CCAAATGGGTGACTGTAGGGTTGAGA
3' HS1	ATTCCCGTTTTTATGAAATCAACTTT
3' 3'HS1	CTCATAGATTTCTCAATGGCCAAA
Oct4	
Name	Primer Sequence 5'-3'
PromoterF1	TGTGCCTTCAGGGGCCAGTC
PromoterF2	AGTCACCCTCTCAGCTCCTCA
PromoterR1	TGGGGTGAAATTTGGCAGGCT
PromoterR2	AGGCTGGGCAGATGGTGCCA
5'EnhF1	CAAAGTCACACTGCACCCGCT
5'EnhF2	ATGTGGCTCCCTCCCATGTAC
5'EnhR1	CACTGGCAAGGATTATCTCATG
5'EnhR2	TGTGTCCAGTTGCCAAATGAGG
DistalEnhF1	CAGGGCACACACACTTTTGCAG
DistalEnhF2	GTATCCAAAAACCCAAGCCAGGTC
DistalEnhR1	TAGCAGGCCCCCAAGGAGGA
DistalEnhR2	ACTGGGAAGGAACTGGCACT

3'EnhF1<br>3'EnhF2<br>3'EnhR1<br>3'EnhR2	TGCCATTACCATCCCACGGT<br>CTAGGGGAGAAGCCCGGGTTG<br>TGGTCCCCACTTCCCCAGGTG<br>GCGGGAACAGGCAGGCTCT
**ChIP qPCR Primers**	
Name	Primer Sequence 5' - 3'
HBB 7F1	CAACAAGGTGCCAAGTCTTTT
HBB 7R1	ACATCACCTGGATGGGACAT
HBB 13F1	GAATGGCCCTAGTCTGGGTA
HBB 13R1	TGCTGCTTTTGAAACAAATGA
HBB 10F1	CCTATGGCAAAAATGGTGCT
HBB 10R1	CATGCAGTAAACAACCGAACA
C5 1F2	TTTGCCATCTGCCCTGTAAG
C5 1R2	AGTCATGCTGAGGCTTAGGG
C5 6F1	TCAGCTCTGCCTTTCTCCTC
C5 6R1	GCAGACCTTAACTGGCATCC
C5 8F1	CAGTTGCATGCTACCTTAAAGA
C5 8R1	AAGCTGAATCTGCTGCCAAC
DistalHBBF	GCCGTAAAACATGGAAGGAA
DistalHBBR	CCCATTTGCTTATCCTGCAT
3'HBBF	TTCCAGAATCTAGCATCTACCTACC
3'HBBR	TGCTTCTGGCTCTGCAGTTA
HBBF	GCAAGAAAGCGAGCTTAGTGA
HBBR	CAAAGAATTCACCCCACCAG
5'HBBF	CCTCACCACCAACTTCATCC
5'HBBR	GCAACCTCAAACAGACACCA
Proximal PromF	TAGATGGCTCTGCCCTGACT
Proximal PromR	CACTTAGACCTCACCCTGTGG
Distal PromF	TGGGGTAATCAGTGGTGTCA
Distal PromR	TTTTGTTCCCCCAGACACTC
Positive Control1F	ACCCTTCAGCAGTTCCACAC
Positive Control1R	ACCCTTCAGCAGTTCCACAC
Positive Control2F	TGGTATTTTATTCTGAAACACAGAGG
Positive Control2R	GCTTCGGTGTTCAGTGGATT
Negative ControlF	CCCAAATGAGATTATGCCACTG
Negative ControlR	CTATGTTTGTGTTGCAGAGCCC
